# Encapsulation of Cinnamic Acid by Cucurbit[7]uril for Enhancing Photoisomerization

**DOI:** 10.3390/molecules25163702

**Published:** 2020-08-14

**Authors:** Na’il Saleh, Muna S. Bufaroosha, Ziad Moussa, Rukayat Bojesomo, Hebah Al-Amodi, Asia Al-Ahdal

**Affiliations:** Department of Chemistry, College of Science, United Arab Emirates University, P.O. Box 15551 Al Ain, UAE; Muna.Bufaroosha@uaeu.ac.ae (M.S.B.); zmoussa@uaeu.ac.ae (Z.M.); 201870166@uaeu.ac.ae (R.B.); 201350031@uaeu.ac.ae (H.A.-A.); asiaamer_kk@icloud.com (A.A.-A.)

**Keywords:** *cis*-cinnamic acid, cucurbituril, photoisomerization, responsiveness, light stimuli

## Abstract

*Cis*- or *Z*-configuration is required for the plant growth-promoting activity of cinnamic acid (CA), whereas the *E*-form is inactive. Herein, we describe the encapsulation of *E*-CA by cucurbit[7]uril (CB7) and show that photoisomerization reactions can be more efficiently controlled in aqueous solutions by utilizing this supramolecular approach. Measurements of UV–visible absorption and proton NMR spectra at different pH values confirm that *E*-CA and its methyl ester, methyl-*E*-cinnamate (MC), form stronger 1:1 host–guest complexes with CB7 compared to cucurbit[8]uril (CB8) or three cyclodextrins (α-, β-, and γ-CD). Irradiation of (300 nm) UV light to an aqueous solution of the CB7-bound *E* isomers induces *E* to *Z* photoisomerization and the dissociation of the complex. When the same solution is irradiated by (254 nm) UV light, *Z* to *E* conformational changes of the unbound *Z* isomers are observed and are accompanied by restoring the host–guest complex formation.

## 1. Introduction

**Scheme 1:**
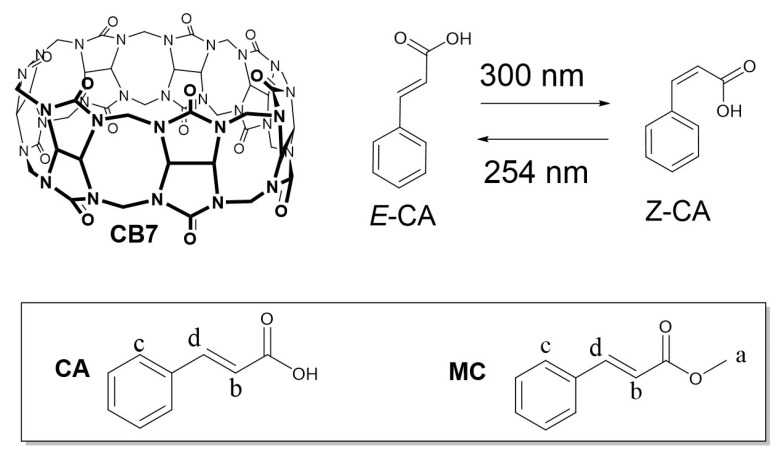
Chemical structures of cinnamic acid (CA), methyl cinnamate (MC), and cucurbit[7]uril (CB7). The photoisomerization equilibrium between the *E* and *Z* forms of CA and the schematic representation of its *E*-CA/CB7 complex are also shown.

Expression of the gene that causes accumulation of cinnamoyl glucose esters during strawberry fruit ripening is negatively regulated by auxin plant hormones and induced by (hydroxyl)cinnamic acids [1]. Recently, it was further concluded that the *cis-* or *Z*-configuration of cinnamic acid (CA) compound is required for its plant growth-promoting activity (Scheme 1) [2]. Methyl-*E*-cinnamate (*E*-MC) is one of the secondary metabolites found in strawberry fruits (Scheme 1) [1]. The *trans-* or *E*-derivatives of CA and MC belong to the class of α,β-unsaturated carbonyl compounds, which reversibly photoisomerize to the *Z* forms (Scheme 1) [3] or undergo photodimerization reactions in aqueous solutions [4]. 

Various supramolecular approaches have been exploited in literature for many years to selectively photodimerize CA [4,5] or to photoisomerize some of its derivatives [6,7]. For example, the noncovalent interactions and the free space offered by various macromolecules such as cucurbiturils (CBs) [8] (Scheme 1) and cyclodextrins (CDs) [9] (Chart S1, Supporting Information) were utilized to orient and control the behavior of the encapsulated guest in the excited state, improving the efficiency of a light-controlled switching between its isomers [6,7].

In general, nonbonded interactions inherently endow the formed host–guest complex with reversible, spontaneous assembly or disassembly, allowing convenient dissociation and reconstruction of the supramolecular system at a low energy cost. Several host–guest systems were reported with applications in food [10,11,12]. However, few specific examples were reported on the implications of the reversible response of CB host–guest systems to chemical stimuli on food technology, which include the use of CB6 to encapsulate 1-methylcyclopropene (1-MCP), an ethylene antagonist, generating a response to sodium bicarbonate and benzoic acid chemical competitors [13], and host–guest complexes of auxin plant hormones to CB7 with a response to pH [14]. 

The employment of synthetic CB host–guest complexes in combination with the photochemistry of various guest molecules has been a popular approach for applications with stimuli-responsiveness [15,16], such as to release/capture of compounds in drug delivery systems [17], to decipher the effects of microenvironment on the emission generated by a biological emitter [18], or to enhance rates of light-activated biological reactions [19], just to name a few. Rare examples were reported on the extension of these light-driven host–guest systems to food technology. An example was the reported host–guest systems of neutral dapoxyl sodium sulfonate with 2-hydroxypropoyl-β-CD for assaying the binding affinities of some stimuli food additives with the host molecule [20].

Herein, we exploit a supramolecular approach to switch on and off the activity of *E*-CA in response to light, which could potentially be deployed during strawberry fruit ripening or other plant growth-promoting activities in the future (Scheme 1). Upon exposure to light of specific wavelengths (300 and 254 nm), *E* to *Z* and *Z* to *E* photoisomerization processes repeatedly occur (Scheme 1). Each form conserves its conformation at ambient conditions because of its thermal stability [3]. Specifically, we present the effects of sequestration of *E*-CA into cucurbit[7]uril (CB7, Scheme 1) on the efficiency of its photoisomerization process. Our results indicate that the interior cavity of CB7 enhances the durability of the transformation between the agrichemical inactive and active forms (*E* and *Z* isomers, respectively) of CA compound. The presented paradigm allows one to better tune the activity of food additives based solely on light control, which paves the way to the fabrication of further nontoxic host–guest complexes with tunable activity for diverse food applications.

## 2. Results and Discussion

### 2.1. Host–Guest Interactions

#### 2.1.1. UV–Visible Absorption Titration (UV)

**Figure 1:**
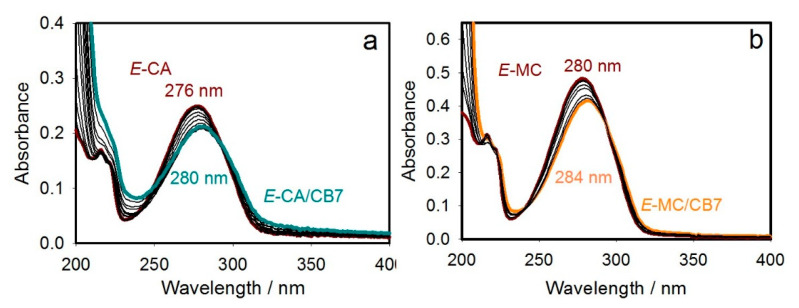
Dependence of the UV–visible absorption spectra of *E*-CA and *E*-MC substrates (18 μM) on the concentration of CB7 (pH 2.5; **a**, and pH 7.4; **b**). For clarity, the initial and final spectra are shown in matching colors with the dominant chemical species. The numbers are the corresponding maxima (in nanometers).

The absorption spectra of *E*-CA and *E*-MC that has an additional methyl group were collected with the addition of CB7 in aqueous solutions (Figure 1). The pH value of *E*-CA’s solution was adjusted to 2.5 for the purpose of investigating the complexation of its neutral forms (*E*-CA) with CB7 and not its anionic form (*E*-C) at pH 7.4 [21]. The host–guest complexation was confirmed by the appearance of an isosbestic point at 290 nm, which also supports the NMR results below in that a 1:1 complex was formed.

**Figure 2:**
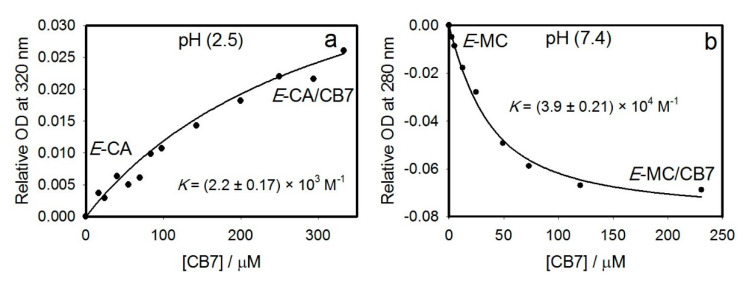
Binding affinities of *E*-CA (**a**) and *E*-MC (**b**) substrates (18 μM) with CB7 (the structures are given in Scheme 1) determined by titration based on UV–visible absorption spectra. The insets show the nonlinear fitting to a 1:1 binding model solid line (Experimental Section). OD is the optical density. Relative OD is the difference between the absorbance in the absence and presence of CB7.

The binding affinities of *E*-CA and *E*-MC with two cucurbiturils (CB7 and CB8) and three cyclodextrins (α-, β-, and γ-CD), whose structures are illustrated in Scheme 1 and Chart S1 (Supporting Information), were extracted from the UV–visible absorption data (Experimental Section). As it is inferred from Table 1 and the complete spectra and titration curves (Figure 2 and Figures S1 and S2, Supporting Information), from the hosts used in this study, *E*-CA and *E*-MC form the strongest complexes with CB7; the interaction with other hosts is weaker or complex does not form. 

Although β-CD has a size similar to that of CB7, it does form a weaker complex with *E*-MC (*K* = 590 ± 20 M^−1^ versus *K* = 39,000 ± 2100 M^−1^), which can be explained by the lack of perfect size matching. The lower binding affinities of other hosts with *E*-MC could also be attributed to their high flexibility, weaker noncovalent interactions, and cavities’ polarity [22]. It must also be noticed that weaker complexation of CB7 to *E*-C species (the anionic form of cinnamic acid) as compared to *E*-CA (the neutral form; *K* = 2200 ± 170 M^−1^) is presumably due to the combined electronic repulsion and steric hindrance exerted by the negatively-charged carboxylate group when positioned in spatial proximity of the carbonyl group of CB7 [6]. The formation of a stable complex between CB7 and *E*-CA is required to ensure its ability to modulate its process of photosimerization, as described below.

#### 2.1.2. NMR Titration (NMR)

**Figure 3:**
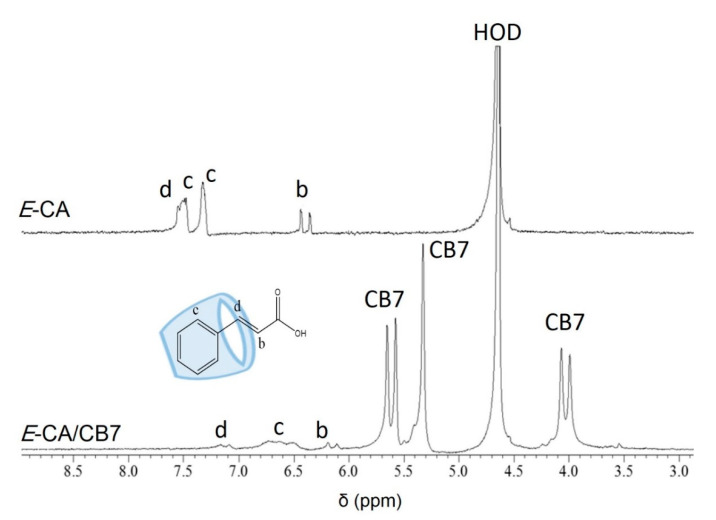
^1^H NMR spectra of *E*-CA (3.25 mM) with CB7 (two equiv.) in D_2_O of pD 2.5 at 298 K (400 MHz). Solvent, guest (b–d), and CB7 peaks are indicated.

NMR titration is normally used in combination with UV titration to confirm the binding affinity, structure, and stoichiometry of the inclusion complex formed. In fact, the chemical shifts (δ) acquired during NMR titration deliver valuable information about the orientation of the encapsulated guest molecule inside the CD cavity, revealing some physical and chemical properties about the host–guest complex [23,24]. In the present work, the formation of host–guest inclusion complex between *E*-CA and CB7 was confirmed by NMR titration at pD 2.5 (Figure 3). Monitoring the proton NMR chemical shifts as a function of the concentrations of CB7 (0–2 equiv.) reveals that *E*-CA has partially entered the cavity of CB7 from the aromatic side. The mode of inclusion was confirmed by the shifts of all aromatic proton resonances H-c to lower ppm (by ~1.1–1.2 ppm) with the addition of CB7, whereas the double bond protons H-b and H-d exhibited much less shift (by ~0.1–0.2 ppm). 

Because *E*-MC possesses an additional site for binding the host, the inclusion of *E*-CA in CB7 was compared to those inclusions of *E*-MC inside two cucurbiturils (CB7 and CB8) and three cyclodextrins (α-, β-, and γ-CD) to confirm our NMR analysis for the inclusion pattern of the *E*-CA/CB7 complex. The corresponding results of ^1^H NMR spectra are depicted in Figures S3–S7, Supporting Information. The mode of inclusion and the orientation of the guest *E*-MC molecules in the cavity of a given host depend on the size of its cavity. For example, α-CD solely and weakly engulfed the methyl group (Figure S5, Supporting Information), whereas the bigger cavity of β-CD accommodated both the methyl and alkene groups (Figure S6, Supporting Information). It also appears that the whole guest molecule was engulfed by the host with the largest cavity size, γ-CD (Figure S7, Supporting Information). CB7 and CB8 have formed complexes with *E*-MC with higher selectively to encapsulate the aromatic group when compared to those of CDs (Figures S3 and S4, Supporting Information).

The corresponding binding affinities were also assessed by applying nonlinear least-square fittings (Experimental Section) to the observed NMR shifts (Table 1 and Figures S8–S11, Supporting Information). The results confirm weaker binding affinities between *E*-CA or *E*-MC and all hosts compared to CB7. Each complex was formed in a 1:1 ratio. Specifically, the Job’s method has confirmed a 1:1 stoichiometry for the inclusion complex of *E*-MC/β-CD (Figure S12, Supporting Information).

Weak binding constants for the association of a series of food additives (including *E*-MC) in α-CD and β-CD were determined using UV–visible titration techniques [25]. Moreover, the assumed geometry of the inclusion complex of *E*-MC with β-CD is based on the crystal structure of complexes of β-CD with *E*-CA [26]. The authors described the *E*-CA encapsulated in the β-CD as a 1:1 complex. They have shown that the phenyl ring is almost perpendicular to the plane of the β-CD. This complex is a dimer where two β-CD molecules encapsulating two *E-CA* acid molecules in their cavities. The X-ray structure of ethyl cinnamate/β-CD complex was also found to be similar to the one of *E*-CA/β-CD complex [27]. The authors attributed this geometry to the polar group (–CH=CH CO_2_Et) of one guest molecule, which becomes extended from the primary hydroxyl-groups in the solid state. The second guest molecule has its polar group (–CH=CH CO_2_Et) extended from the secondary hydroxyl groups. However, they suggested that a dynamic equilibrium might occur.

### 2.2. Stimuli-Responsiveness to Light through the Photoisomerization Process

**Figure 4:**
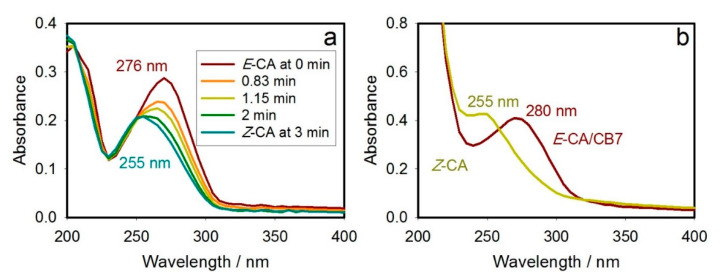
UV–visible absorption spectra of *E*- and *Z*-CA (20 µM) in water at pH 6 and 298 K upon irradiation from a UV (300 nm) lamp for three minutes without (**a**) and with 1 mM CB7 (**b**). For clarity, the initial and final spectra are shown in matching colors with the dominant chemical species (the structures are given in Scheme 1). The numbers are the corresponding maxima (in nanometers) and irradiation time (in minutes).

Irradiation of UV light (300) to a solution of the *E* isomer of CA (Chemical Abstracts Service Registry Number, 140-10-3) in water at pH 6 and 298 K, resulted in its photoismerization to the *Z* form (Scheme 1). The *E* to *Z* photoisomerization of *E*-CA, monitored by UV–visible absorption spectroscopy, resulted in large changes in the absorption profiles of the compound, accompanied by a blue shift (~12 nm) of the absorption maximum in the absence (Figure 4a) or presence of CB7 (Figure 4b). Table 2 summarizes the corresponding absorption maxima and molar absorptivity values associated with the π–π * absorption bands of both *E* and *Z* isomers in the same media.

**Figure 5:**
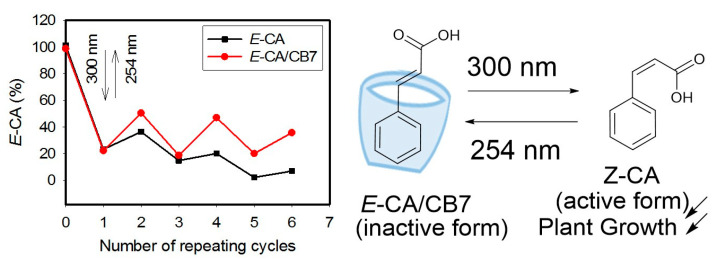
Percentage changes for *E*-CA upon repeated 300 nm (descending lines) and 254 nm (ascending lines) irradiation in water at pH 6 and 298 K in the absence (bottom, black lines) and presence of CB7 (top, red lines).

The formation of stable *E*-CA/CB7 complex in aqueous solution suggests the use of CB7 barrel to repeatedly switch the photoisomerization of *E*-CA. The measurements were carried out at pH 6, and the results indicated that the new host–guest complex has a photoswitchable “ on–off ” property [6]. While similar blue-shift was observed upon irradiation of UV (300 nm) to the aqueous solution of *E*-CA/CB7 (Figure 4b) as for the photoisomerization of free *E*-CA (Figure 4a), more pronounced changes in the UV–visible absorption spectra of *E*-CA/CB7 were achieved upon the repeated irradiation of two UV lights (300 and 254 nm, respectively) to an aqueous solution of the complex compared to the observed plot in the absence of CB7 (Figures S13 and S14, Supporting Information). However, changes in the absorbance alone does not confirm that there is a higher photoisomerization efficiency for *E*-CA/CB7 than for free *E-*CA as these systems apparently possess different extinction coefficients. Thus, rigorous quantitation of the *E* and *Z* forms was conducted by NMR measurements (see below) to calculate the conversion in Figure 5.

The maximum *E* to *Z* or *Z* to *E* ratios were achieved at 3 min when the complex was exposed to irradiation at 300 and 254 nm, respectively. Thus, 3 minutes of irradiation time was also used when investigating the corresponding photoisomerization properties of the free *E*-CA. The similarity of the two spectra corresponded to the Z species in Figure 4a,b indicates that UV irradiation caused dissociation of the *Z*-CA/CB7 complex (see also NMR results). More importantly, the repeated irradiation for 3 min of 300 and 254 nm light confirmed higher photoisomerization efficiency in the CB7-complexed *E*-CA compared to that isomerization in the free *E*-CA. It must be said that at pH 6, a considerable proportion of CA molecules may exist in their anionic form [21]; thus, while the phenyl ring of *E*-CA is included in the CB7’s cavity (Figure S15, Supporting Information), the carboxylate group remains positioned outside the cavity of CB7. Kim et al. encapsulated some cinnamamide derivatives in CB7 and observed that only the *E*-form was included. The results were rationalized by the spatial proximity of the negatively-charged carboxylate group attached to the amide terminal of cinnamamide [6]. 

### 2.3. Quantitation of Formed Photoisomerization Product Ratios

As a second piece of spectroscopic evidence on the state of the system at each step in the photoisomerization and to provide a clear experimental evidence that the *Z*-guest isomer is not bound by CB7 (Figure 5), NMR spectra of both the free and CB7-complexed alkene were measured with increasing alternated irradiations of UV lights (300 and 254 nm). Changes in the ^1^H NMR spectra of *E*-CA (1.6 mM in D_2_O, pD 6) were monitored with increasing irradiation of UV light (300 nm) up to 10 min. The resonance of protons d and b corresponded to the alkene group of the *E* isomer, which shifted to a lower ppm with a concomitant increase in the ratio of *Z* to *E* isomers up to a maximum (*Z*-CA:*E*-CA = 9:1) at 5 min (Figure S16 and Table S1, Supporting Information). The measurements upon irradiation of UV light (254 nm) of that final solution was conducted up to 10 min to confirm that the photoisomerization is reversible with no degradation because the *E* alkene is also present at the end of the experiment (Figure S17 and Table S2, Supporting Information). Assignments of protons in the alkene were confirmed by observing a cross peak in its COSY spectrum and from the coupling constants between H-d and H-b (16 Hz for the *E* isomer and 12 Hz for the *Z* isomer). The results also reveal the overlap of the d proton from the *Z* isomer with the b proton from the *E* isomer at 6.3 ppm (Figure S18, Supporting Information).

^1^H NMR spectra of *E*-CA (1.62 mM) were also measured following irradiation with UV light (300 nm) at 5 min in the presence of approximately two equivalents of CB7 (Figure S19, Supporting Information). The resulting mixture of *E*-CA and *Z*-CA isomers were then exposed to UV light (254 nm) for 10 min (Figure S20, Supporting Information). The stacked ^1^H NMR spectra in Figures S19 and S20, Supporting Information, confirm the dissociation of the Z-CA/CB7 complex. Because those NMR peaks were too broad, the isomeric ratio could not be measured by integration. However, a previously reported method was used to calculate the conversion in Figure 5 with rigorous quantitation of the *E* and *Z* forms (Tables S3 and S4 and Figure S21, Supporting Information) [6]. The results confirm that the yield of *Z* alkene is indeed enhanced in the presence of CB7.

Based on the presented data, in Figure 5, it appears that the photoisomerization is more efficient in the presence of CB7. It is not clear whether this improvement in the light fatigue resistance of the guest when encapsulated by CB7 is due to an increase in the absorbance at 300 nm of the CB7-complexed *E*–alkene (Figure 1a), protection of the *E*-alkene from photodegradation by encapsulation, some other changes in the photophysical properties of *E*-CA due to encapsulation in CB7, or some other supramolecular catalytic effects. Nonetheless, it appears that the amount of the *E*-alkene formed from photoisomerization using 254 nm light is improved in the presence of CB7 (Figure 5). Perhaps this is because there is more *Z* isomer present in the sample with CB7.

After multiple cycles of photoisomerization, the amount of the *E* isomer is 50% restored (Figure 5), because the complex sustained more the switching process compared to the unbound *E*-CA, signifying an improved transformation efficiency, which has potential value in developing reversible responsiveness systems. Faster reversibility of the host–guest system in response to light should have potential implications for switching on and off its plant-growth-promoting activity in the future. CBs have formed more stable complexes with *E*-CA and *E*-MC with higher selectively to encapsulate the aromatic group when compared to those of CDs. These results highlight their superiority when it comes to their applications in developing stimuli-responsive activity in food science. The implication of stimuli-responsive supramolecular systems in controlling the photoactivation of food additives has rarely been reported in the literature.

## 3. Experimental 

### 3.1. Materials

CB7, CB8, α-CD, β-CD, γ-CD, *E*-cinnamic acid (*E*-CA), methyl-*E*-cinnamate (*E*-MC), and the deuterated solvent (D_2_O) were purchased from Sigma-Aldrich (St. Louis, MO, USA) (purity > 99%) and used as received without any further purification. Deionized water (Millipore water with conductivity less than 0.05 μs) was used in optical measurements. The temperature was kept at 298 K for all the experiments. 

### 3.2. ^1^H NMR Titration

^1^H NMR spectra were recorded using Varian 400 MHz spectrometer (Varian, Palo Alto, CA, USA) in D_2_O by using tetramethylsilane (TMS) as an internal reference. In the NMR titration, calculated weights of host molecule were gradually added to a stock solution of free guest molecule prepared in D_2_O (1 mL). NMR measurements were taken immediately after each addition of host. As for Job’s plot, various calculated weights of both guest (G) and host (H) were added to a fixed volume of D_2_O, resulting in different ratio of concentrations while maintaining a fixed total concentration for the two components.

### 3.3. Optical Measurements

The UV–visible absorption spectra were measured using Varian UV–visible Cary-50 spectrophotometer (Agilent Technologies, Santa Clara, CA, USA) at 298 K. The instrument has a 0.2 nm resolution and 300 nm/min scan speed. The measurements were carried out in a rectangular cuvette (1 cm optical path length) from Starna (Atascadero, CA, USA). The pH (pD) was adjusted by adding aliquots amount of NaOH (NaOD) or HCl (DCl). Readings were taken with a WTW 330i pH meter equipped with a combined pH glass electrode (Sen Tix Mic).

### 3.4. Determination of Binding Affinities 

The binding constants (*K*) were extracted from the changes of UV–visible absorption spectra and NMR resonances of G with the addition of different amounts of H at a given pH (pD). The measurements were carried out before and after adding small amounts of H’s stock solution to a known concentration of G using an automatic pipette until a plateau was reached. While the total concentrations of G were kept constant, the concentrations of H were gradually increased. The analysis of the resulting spectra involves the assumption of a 1:1 equilibrium between the agrochemical compounds and the host molecules, as follows:

(1) G+H⇌G/H

where K is the association equilibrium constant:

(2) $$K=\frac{\left[G/H\right]}{\left[G\right]\left[H\right]\;\;\;}$$

Using the laws of mass balance:

*C*_G_ = [G] + [G/H] (3)

*C*_H_ = [H] + [G/H] (4)

where *C*_G_ and *C*_H_ are the total concentrations of G and H, respectively. The Y symbol refers to the optical density (OD) from the UV–visible absorption spectral measurements or the chemical shifts (δ) from the NMR measurements of G. This gives for the case of optical analysis:

Y^λ^=*ε*_G_ [G] + *ε*_G/H_ [G/H] (5)

where *ε*_G_ and *ε*_G/H_ are the molar extinction coefficients (M^–1^ cm^–1^) of the free and H-complexed chemical form, respectively, of G. A combination of Equations (1)–(4) gives

(6) $$\Delta Y^{\lambda }=\frac{\left(\Delta \varepsilon \right)C_{H}}{\frac{2}{KC_{G}-1-KC_{H}+\sqrt{{\left(1-KC_{G}+KC_{H}\right)}^{2}+4KC_{G}}}+1}$$

where Δ*Y*^λ^ is the spectral changes at a given *λ* as a function of H concentration, Δ*ε* is the difference between the molar extinction coefficients of uncomplexed and complexed G, and *K* is the binding constant. The binding constants (*K*) in Table 1 were then obtained by using the nonlinear formula of Equation (5), utilizing Levenberg-Marquardt algorithm which is available in SigmaPlot software (version 6.1; SPCC, Inc., Chicago, Illinois, USA). The molar extinction coefficient of the complex was left as a floating parameter in the analysis.

### 3.5. Photoisomerization Measurements 

Changes in the UV–visible absorbance of an aqueous solution of the free and complexed *E*-CA at pH 6 were monitored upon irradiation in a Luzchem LZC-4V photoreactor (Ottawa, ON, Canada) equipped with fluorescent light tubes; UVB lamps (300 nm) and UVC lamps (254 nm). Power/intensity of UVB and UVC lamps were measured to be 18 and 12 mW/cm^2^, respectively.

## 4. Conclusions

The reversibility and responsiveness of *E*-CA/CB7 supramolecular host–guest system in aqueous solutions have been best utilized to develop a highly reversible and efficient response of *E*-CA to light, potentially generating a light-driven control of its plant-growth-promoting activity. A stable complex in a 1:1 ratio was formed upon the sequestration of the *E* isomer of CA (inactive form) into CB7. The complex dissociated to its *Z* isomer (active form) when it was irradiated with 300 nm light as confirmed by UV–visible and ^1^H NMR spectroscopy. Consequently, when the *Z* isomer was exposed to irradiation of 254 nm light, the opposite process happened, restoring the complexation. The dissociation of the guest from CB7 occurred because the two negatively charged carbonyls of the guest and CB7 portal, in spatial proximity, repel one another.

## Figures and Tables

**Table 1:** Binding constants of *E*-CA, *E*-C, and *E*-MC compounds with different macrocyclic hosts as determined by UV–visible absorption titration (UV) and NMR titration (NMR).

Substrates	CB7	CB8	α-CD	β-CD	γ-CD
*E*-CA	(2.2 ± 0.2) × 10^3^ M^−1^ (UV) (2.6 ± 1.2) × 10^3^ M^−1^ (NMR)	ND *^a^*	ND *^a^*	ND *^a^*	ND *^a^*
*E*-MC	(3.9 ± 0.2) × 10^4^ M^−1^ (UV)	NF ^b^	(1.9 ± 0.1) × 10^3^ M^−1^ (UV) (3.5 ± 0.2) × 10^2^ M^−1^ (NMR)	(5.9 ± 0.2) × 10^2^ M^−1^ (UV) (1.4 ± 0.6) × 10^2^ M^−1^ (NMR)	(1.7 ± 0.2) × 10^2^ M^−1^ (NMR)
*E*-C ^c^	NF ^b^	ND *^a^*	ND *^a^*	ND *^a^*	ND *^a^*

*^a^* Not determined. *^b^* Complex does not form or host–guest interaction is very weak. ^c^ *E*-C is the anionic form of *E*-cinnamic acid.

**Table 2:** Absorption features of *E* and *Z* isomers of CA in water at pH 6 and 298 K.

Chemical Form	Max *λ*_abs_/nm for Given Form (ε/M^−1^cm^−1^)
*E*-CA	276 (13,835)
*Z*-CA	255 (10,370)
*E*-CA/CB7	280 (10,635)

## Supplementary Materials

The following are available online, Chart S1: The structures of the tested cyclodextrins macrocycles and cucurbit[8]uril (CB8) that were tested as hosts in this study; Figure S1. Binding affinities of the anionic form of *E*-cinnamic acid (*E*-C with CB7; a), and *E*-MC (with CB8, α-CD, and β-CD; b, c, and d) at a concentration of 20 μM and pH 7.4 (the structures are given in Scheme 1 and Chart S1) determined by titration based on UV–visible absorption spectra. The *insets* show the nonlinear fitting to a 1:1 binding model solid line (Experimental Section). OD is the optical density. Relative OD is the difference between the absorbance in the absence and presence of the macrocycle; Figure S2. Dependence of the UV–visible absorption spectra of the anionic form *E*-cinnamic acid (*E*-C with CB7; a) and *E*-MC (with CB8, α-CD, and β-CD; b, c, and d) at a concentration of 20 μM (the structures are given in Scheme 1 and Chart S1). For clarity, the initial and final spectra are shown in matching colors with the dominant chemical species. The numbers are the corresponding maxima (in nanometers); Figure S3. ^1^H NMR spectra (400 MHz) of *E*-MC (0.5 mM) with CB7 (0.5 mM) in D_2_O at pD 7; Figure S4. ^1^H NMR spectra (400 MHz) of *E*-MC (0.5 mM) with CB8 (0.5 mM) in D_2_O at pD 7; Figure S5. ^1^H NMR spectra (400 MHz) of *E*-MC (0.5 mM) with α-CD (19.7 mM) in D_2_O at pD 7; Figure S6. ^1^H NMR spectra (400 MHz) of *E*-MC (0.6 mM) with β-CD (6.8 mM) in D_2_O at pD 7; Figure S7. ^1^H NMR spectra (400 MHz) of *E*-MC (0.38 mM) with γ-CD (11.7 mM) in D_2_O at pD 7; Figure S8. ^1^H NMR (400 MHz) titration of (a) *E*-CA (3.25 mM) with CB7 at pD 2.5, (b) *E*-MC (0.5 mM) with α-CD at pD 7, (c) *E*-MC (0.6 mM) with β-CD, and (c) *E*-MC (0.38 mM) with γ-CD at pD 7 in D_2_O. Nonlinear fitting plots (Experimental Section) of chemical shift (δ; ppm) versus concentration of the macrocycles in molarity (M) for the extraction of binding affinities (*K*) are shown (R =0.99). The monitored NMR peak is also indicated; Figure S9. ^1^H NMR (400 MHz) titration of *E*-MC (0.5 mM) with α-CD (0–37 equivalents) in D_2_O at pD 7; Figure S10. ^1^H NMR (400 MHz) titration of *E*-MC (0.6 mM) with β-CD (0–15 equivalents) in D_2_O at pD 7; Figure S11. ^1^H NMR (400 MHz) titration of *E*-CA (3.25 mM) with CB7 (0–2.34 equivalents) in D_2_O at pD 2.5; Figure S12. (A) ^1^H NMR spectra (400MHz, 2 mM = [MC] + [β-CD]), from which the peak at 7.54 ppm in the bottom spectra was monitored; and (B) Job Plot constructed from the data in part (A); Figure S13. Absorption spectra upon repeated exposure of UV light (300 and 254 nm) to an aqueous solution of CA (16 µM) at pH 5.5 and 298 K as a function of exposure time (each isomer was exposed to irradiation for 3 min at each run) in the absence of CB7; Figure S14. Absorption spectra upon repeated exposure of UV light (300 and 254 nm) to an aqueous solution of CA (32 µM) at pH 5.8 and 298 K as a function of exposure time (each isomer was exposed to irradiation for 3 min at each run) in the presence of CB7 at 1 mM concentration; Figure S15. ^1^H NMR spectra of *E*-CA (1.62 mM) with CB7 (0–2 equivalents) in D_2_O (pD 6) at 298 K (400 MHz). Solvent and CB7 peaks are indicated; Figure S16. ^1^H NMR spectra of *E*-CA (1.62 mM) in D_2_O (pD 6) at 298 K before and after irradiation with UV light (300 nm) up to 10 min (400 MHz). Solvent peak is indicated; Figure S17. ^1^H NMR spectra of a 9:1 mixture of *Z*-CA and *E*-CA (total concentration of 1.62 mM) in D_2_O (pD 6) at 298 K before and after irradiation with UV light (254 nm) up to 10 min (400 MHz). Solvent peak is indicated; Figure S18. ^1^H NMR COSY spectrum of a 9:1 mixture of *Z*-CA and *E*-CA (total concentration of 1.62 mM) in D_2_O (pD 6) at 298 K (400 MHz); Figure S19. ^1^H NMR spectra of (a) *E*-CA (1.62 mM), (b) after irradiation of UV light (300 nm) to (a) for 5 min, (c) *E*-CA/CB7 (1.62 mM for *E*-CA and 3.46 mM for CB7), and (d) after irradiation of UV light (300 nm) to (b) for 5 min in D_2_O (pD 6) at 298 K (400 MHz). Solvent and CB7 peaks are indicated; Figure S20. ^1^H NMR spectra of (a) a 9:1 mixture of *Z*-CA and *E*-CA (total concentration of 1.62 mM), (b) after irradiation of UV light (254 nm) to (a) for 10 min, (c) a mixture of Z-CA and *E*-CA/CB7 (total concentration of 1.62 mM for CA and 3.46 mM for CB7), and (d) after irradiation of UV light (254 nm) to (b) for 10 min in D_2_O (pD 6) at 298 K (400 MHz). Solvent and CB7 peaks are indicated; Figure S21 Calibration curves for (a) *E*-CA, and (b) *E*-CA/CB7 complex plotted using the values in Table S3; Table S1. The measured percentages associated with the *E* to *Z* photoisomerization upon irradiation of 300 nm to a solution of *E*-CA (1.62 mM) in D_2_O at pD 6; Table S2. The measured percentages associated with the *Z* to *E* photoisomerization upon irradiation of 254 nm to a mixture of *Z*-CA isomer and *E*-CA (*Z*-CA:*E*-CA = 9:1) isomer in D_2_O at pD 6; Table S3. Absorbances (Abs.) data of *E*-CA (16 µM in water and 32 µM in CB7) at 276 nm in the absence and presence of CB7 (1 mM) associated with the *E* to *Z* photoisomerization upon irradiation of UV light (300 nm); Table S4. The calculated percentages of *E*-CA and *E*-CA/CB7 associated with alternating irradiation of 300 nm (3 min) and 254 nm (3 min) from the absorbances (Abs.) data in Figures S13 and S14.
