# Supplementary material for: Encapsulation of Cinnamic Acid by Cucurbit[7]uril for Enhancing Photoisomerization
Source: Molecules. 2020 Aug 14;25(16):3702. doi: 10.3390/molecules25163702 (PMC7464550; doi:10.3390/molecules25163702)
Supplement: Supplementary file 1 [file molecules-25-03702-s001.pdf]

SUPPORTING INFORMATION

Encapsulation of Cinnamic Acid by Cucurbit[7]uril for Enhancing Photoisomerization in Aqueous Solutions

Na'il Saleh,* Muna S. Bufaroosha, Ziad Moussa, Rukayat Bojesomo, Hebah Al-Amodi, and Asia Al-Ahdal

Department of Chemistry, College of Science, United Arab Emirates University, P.O. Box 15551, Al Ain, United Arab Emirates

TABLE OF CONTENTS

Part I: Titrations……………………………………………………………………..………………S3

Binding affinities of *E*-C with CB7 and *E*-MC with CB8, α-CD and β-CD ……………..……S4

Absorption spectra of *E*-C with CB7 and *E*-MC with CB8, α-CD and β-CD……………..…..S5

Part II: Binding Studies by NMR Spectroscopy ………………………………………….....…S6

Binding of MC with CB7 ……………………………………………………………...…S6

Binding of MC with CB8 ……………………………………………………………...…S7

Binding of MC with α-CD ………………………………………………………………S8

Binding of MC with β-CD ……………………………………………...…………….…S9

Binding of MC with γ-CD ………………………………………………….………..…. S10

Nonlinear fitting plots for the binding affinities …………………………………....…. S11

Part II: Binding Titration by NMR Spectroscopy …..…. S12

Titration of CA with α-CD ……………………………………………………..………S12

Titration of CA with β-CD ……………………………………………..…………S13

Titration of CA with CB7 ……………………………………………………...………S14

Titration of MC with β-CD and Job's plot.….……………………………………….…S15

Part III: Interactions CA with UV light with time variance ……………………….............S16

Absorption spectra of CA in the presence of UV light at different times ……………...…S16

Absorption spectra of CA with CB7 in the presence of UV light at different times …..…S17

Part IV: NMR experiments……………………………………………………..……..………S19

NMR binding titration at pD 6……………………………………………………………….S19

NMR spectra of CA in the presence of UV light at different times …………………...….S19-S23

NMR spectra of CA with CB7 in the presence of UV light at different times ………...…S24-25

Calibration curves for calculating *E*/*Z* ratios…………………………………….…..…S26-28

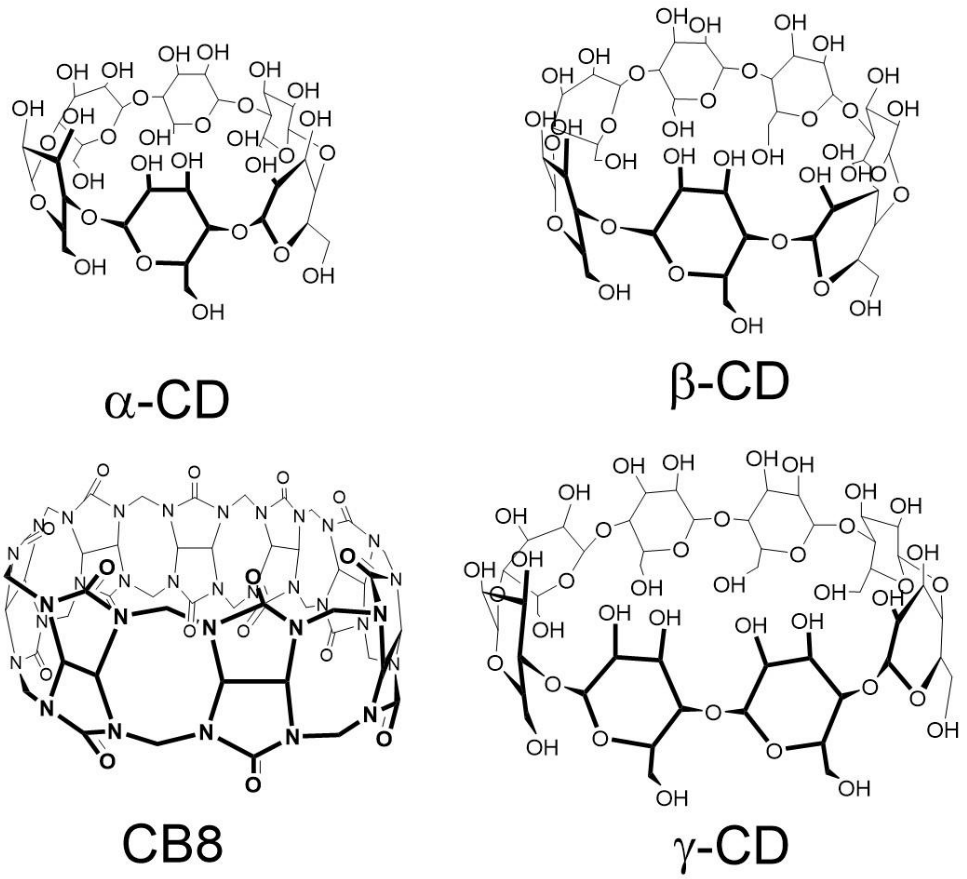

Chart S1. The structures of the tested cyclodextrins macrocycles and cucurbit[8]uril (CB8) that were tested as hosts in this study.

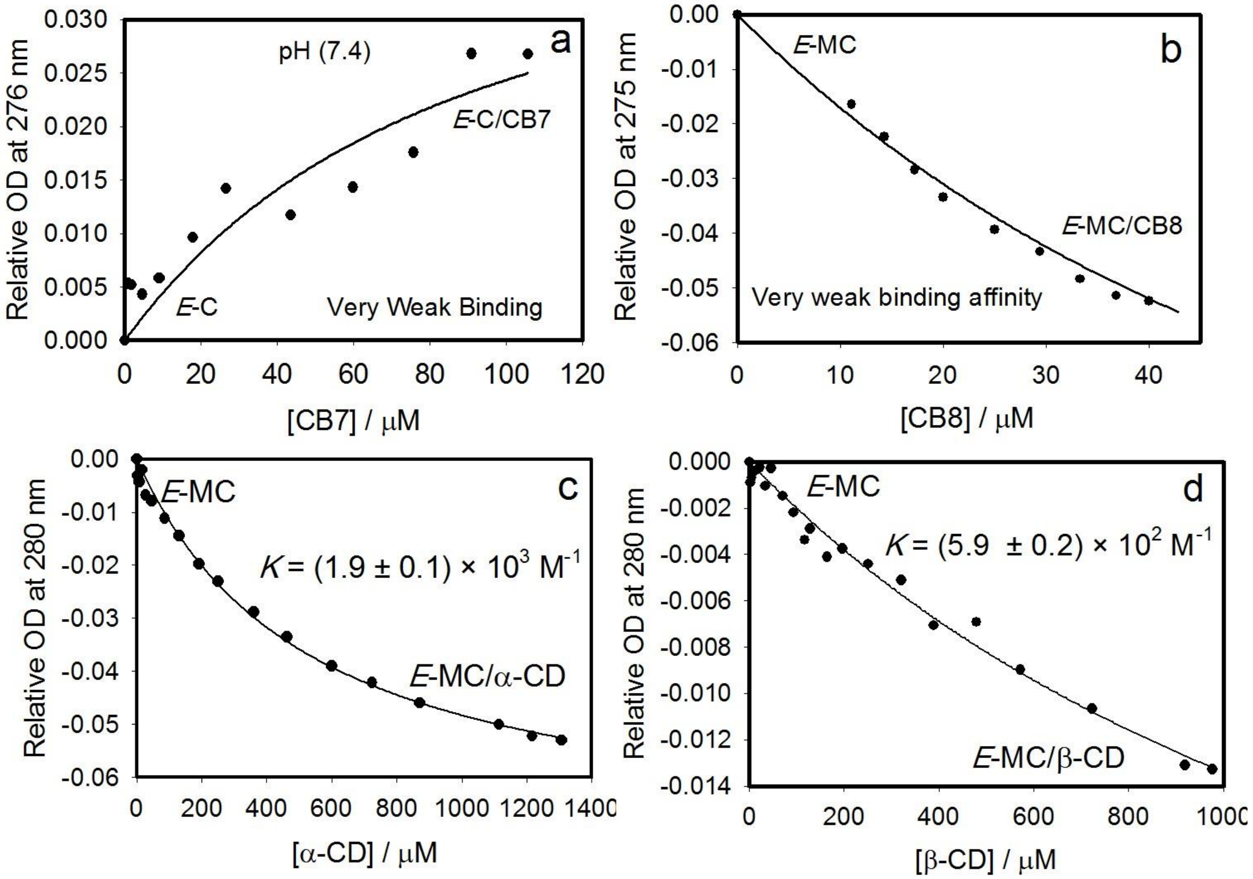

Figure S1. Binding affinities of the anionic form of *E*-cinnamic acid (*E*-C with CB7; a), and *E*-MC (with CB8, α-CD, and β-CD; b, c, and d) at a concentration of 20 μM and pH 7.4 (the structures are given in Chart 1 and Chat S1) determined by titration based on UV−visible absorption spectra. The *insets* show the nonlinear fitting to a 1:1 binding model solid line (Experimental Section). OD is the optical density. Relative OD is the difference between the absorbance in the absence and presence of the macrocycle.

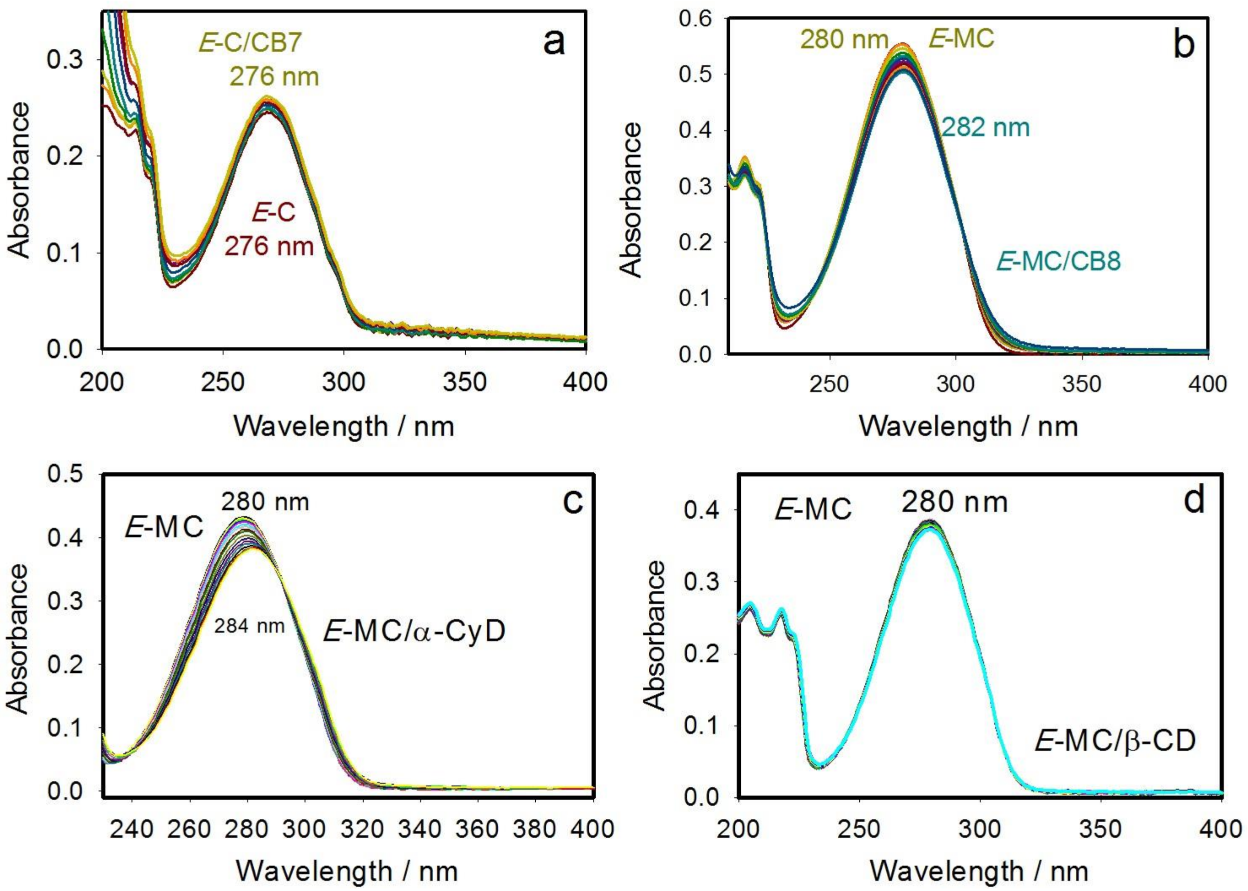

Figure S2. Dependence of the UV−visible absorption spectra of the anionic form *E*-cinnamic acid (*E*-C with CB7; a) and *E*-MC (with CB8, α-CD, and β-CD; b, c, and d) at a concentration of 20 μM (the structures are given in Chart 1 and Chat S1). For clarity, the initial and final spectra are shown in matching colors with the dominant chemical species. The numbers are the corresponding maxima (in nanometers).

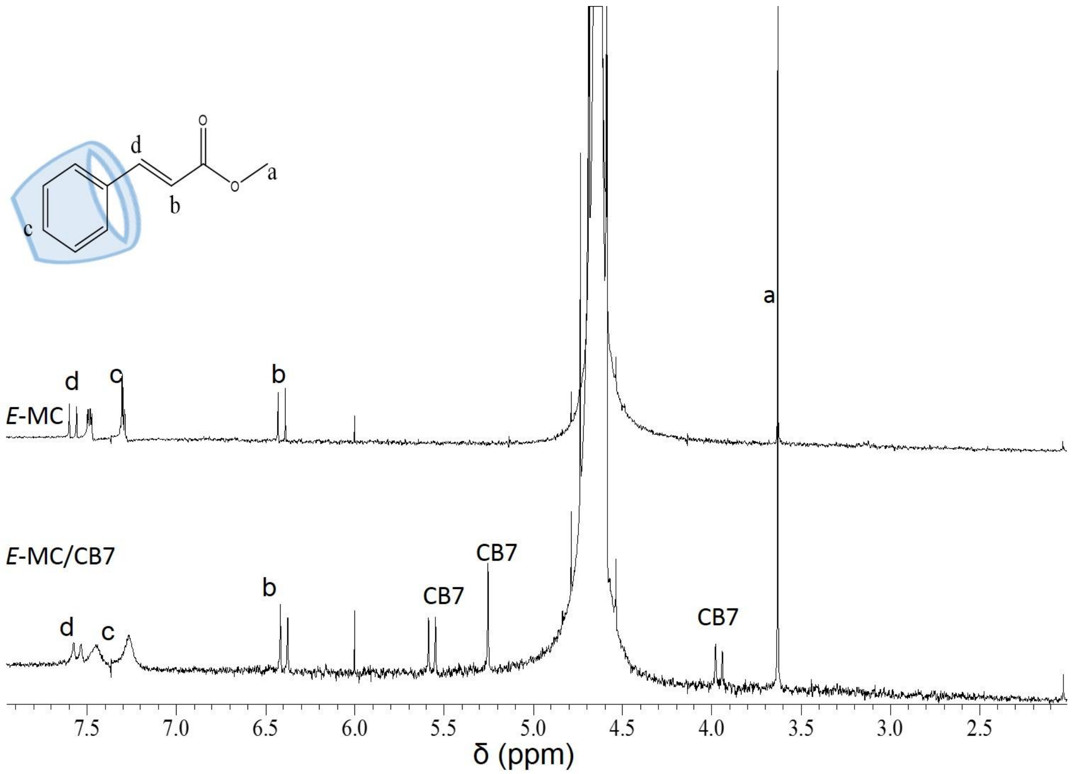

Figure S3. ^{1}H NMR spectra (400 MHz) of *E*-MC **(**0.5 mM) with CB7 (0.5 mM) in D_2O at pD 7.

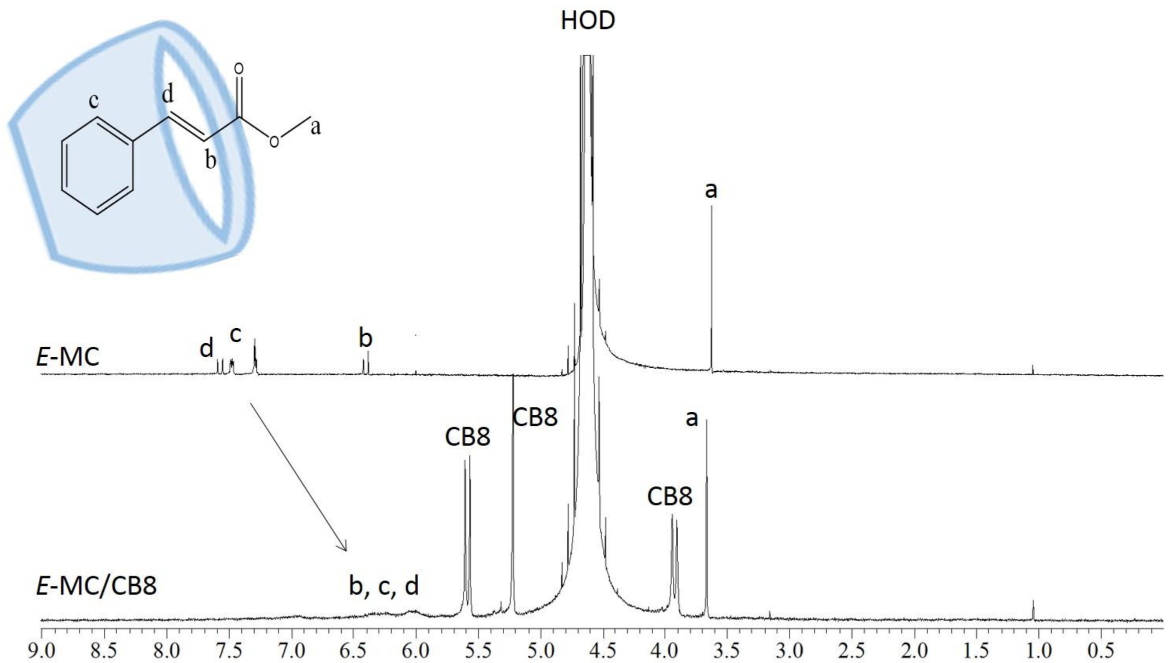

Figure S4. ^{1}H NMR spectra (400 MHz) of *E*-MC (0.5 mM) with CB8 (0.5 mM) in D_2O at pD 7.

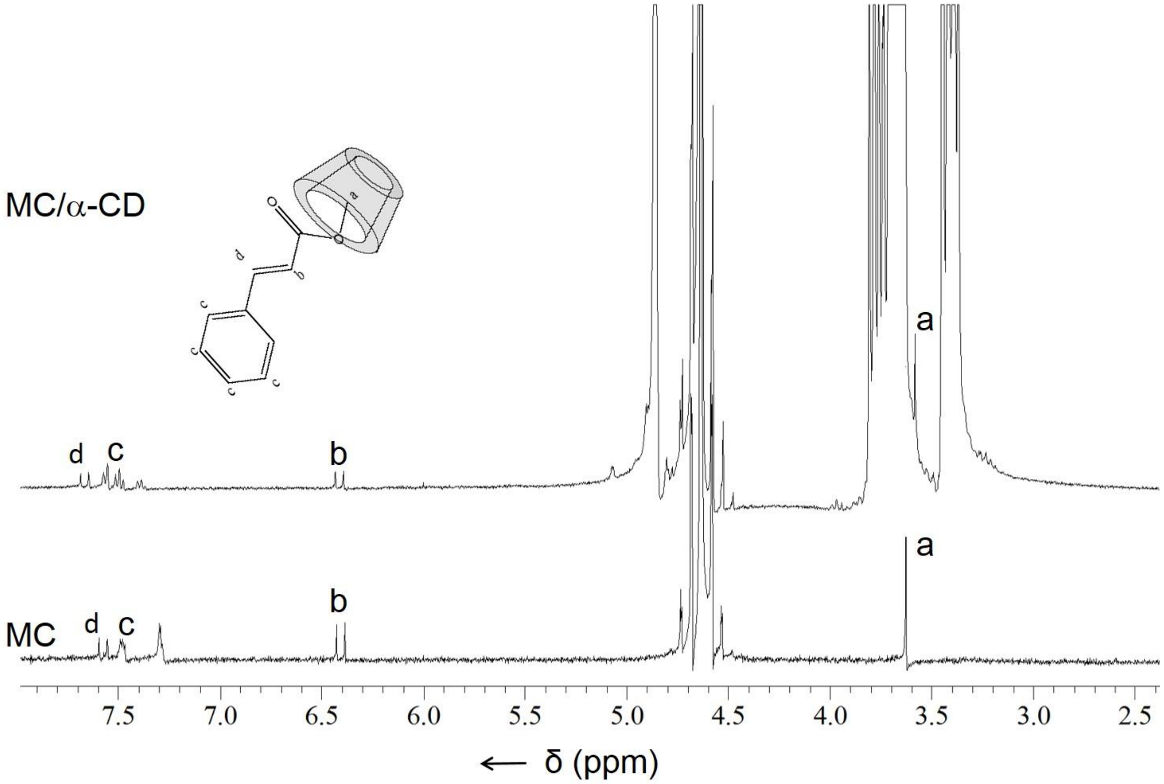

Figure S5. ^{1}H NMR spectra (400 MHz) of *E*-MC **(**0.5 mM) with α-CD (19.7 mM) in D_2O at pD 7.

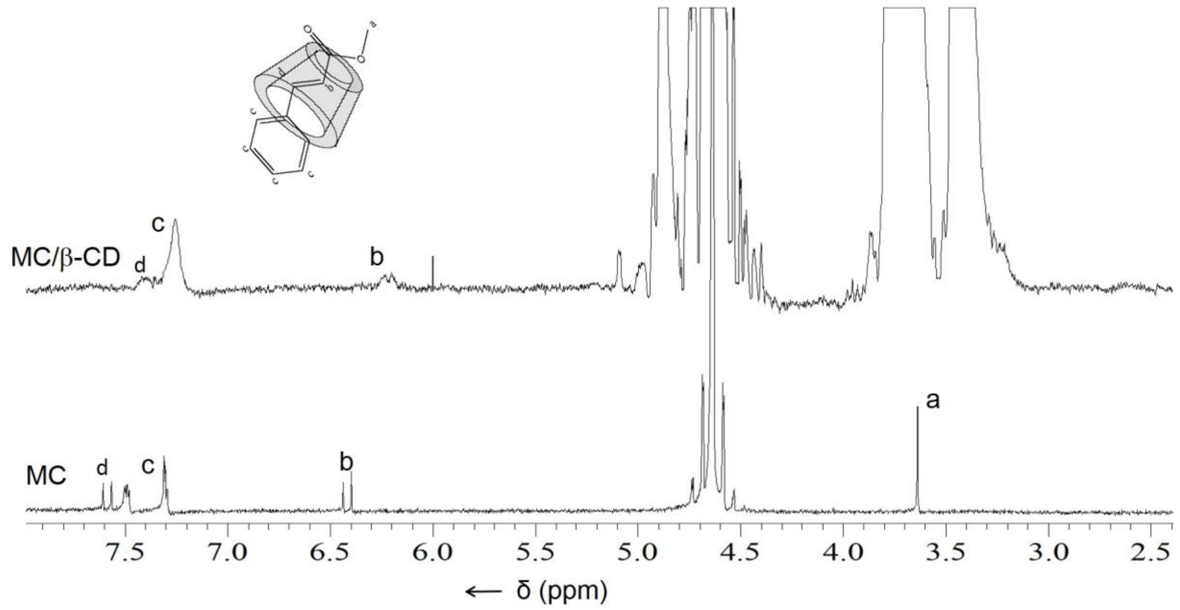

Figure S6. ^{1}H NMR spectra (400 MHz) of *E*-MC **(**0.6 mM) with β-CD (6.8 mM) in D_2O at pD 7.

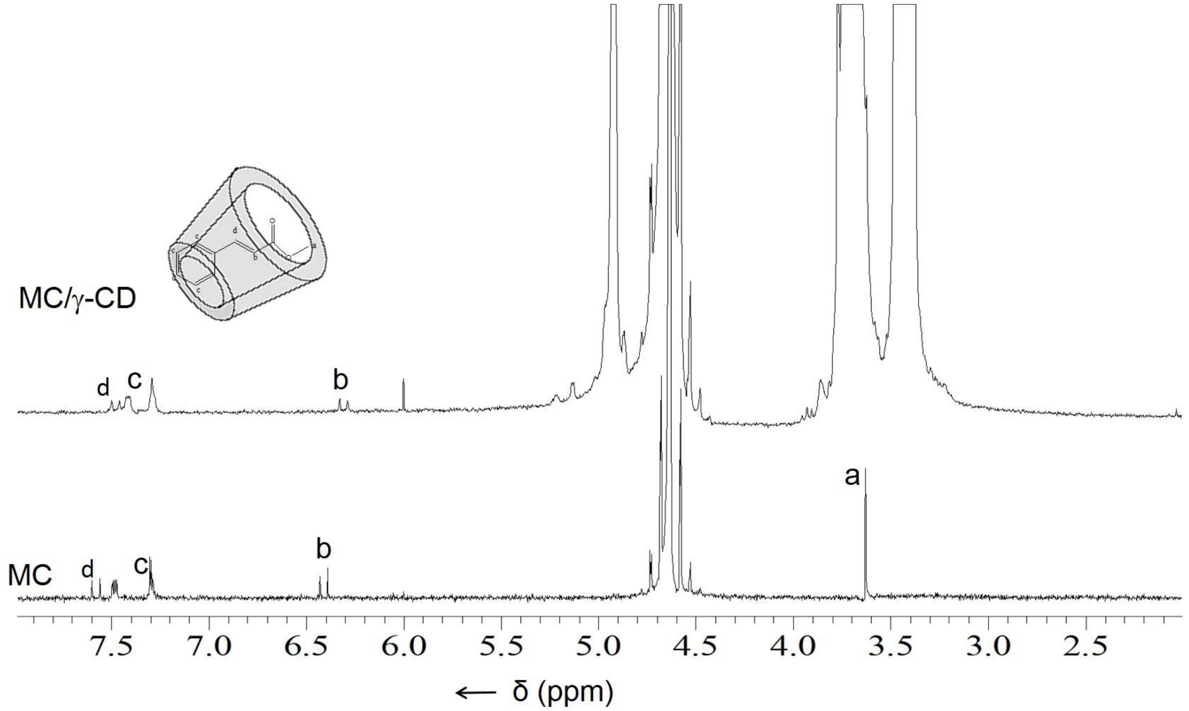

Figure S7. ^{1}H NMR spectra (400 MHz) of *E*-MC (0.38 mM) with γ-CD (11.7 mM) in D_2O at pD 7.

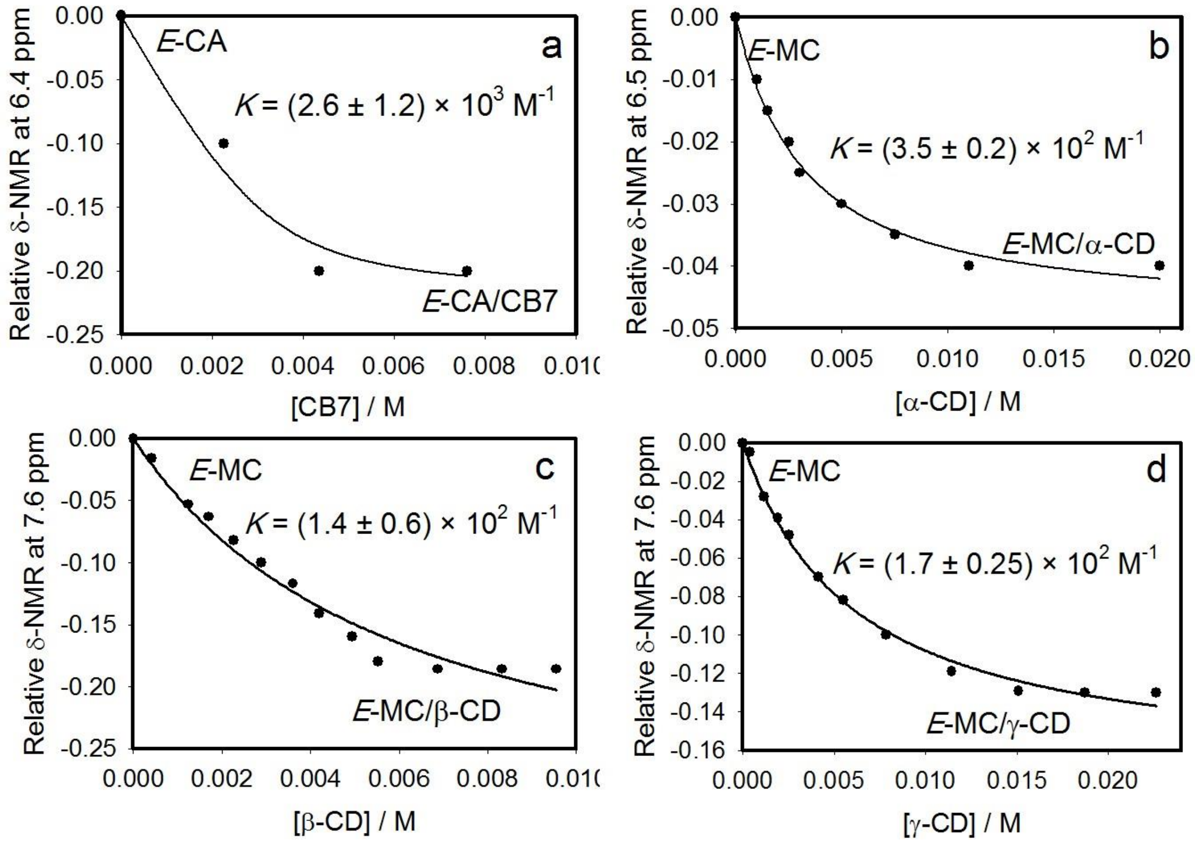

Figure S8. ^{1}H NMR (400 MHz) titration of (a) *E*-CA (3.25 mM) with CB7 at pD 2.5, (b) *E*-MC **(**0.5 mM) with α-CD at pD 7, (c) *E*-MC **(**0.6 mM) with β-CD, and (c) *E*-MC **(**0.38 mM) with γ-CD at pD 7 in D_2O. Nonlinear fitting plots (Experimental Section) of chemical shift (δ; ppm) versus concentration of the macrocycles in molarity (M) for the extraction of binding affinities (*K*) are shown (R =0.99). The monitored NMR peak is also indicated.

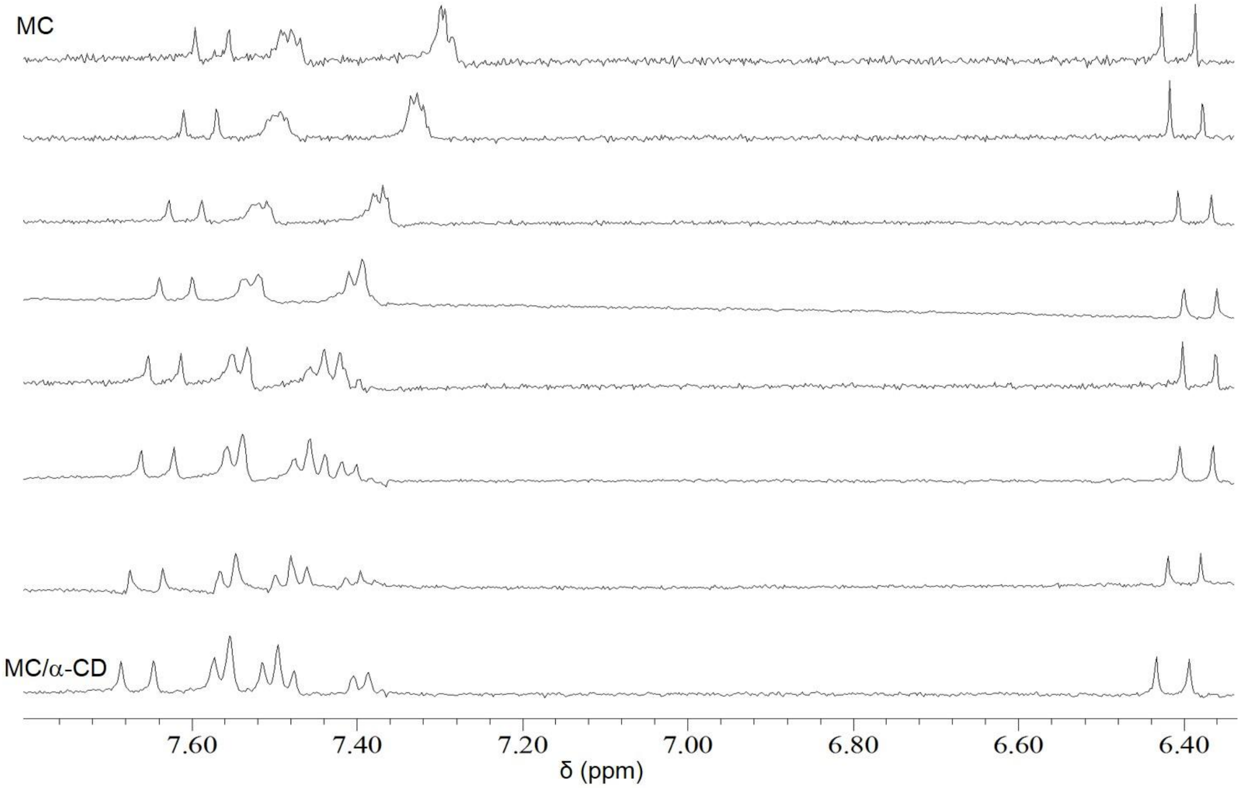

Figure S9. ^{1}H NMR (400 MHz) titration of *E*-MC (0.5 mM) with α-CD (0−37 equivalents) in D_2O at pD 7.

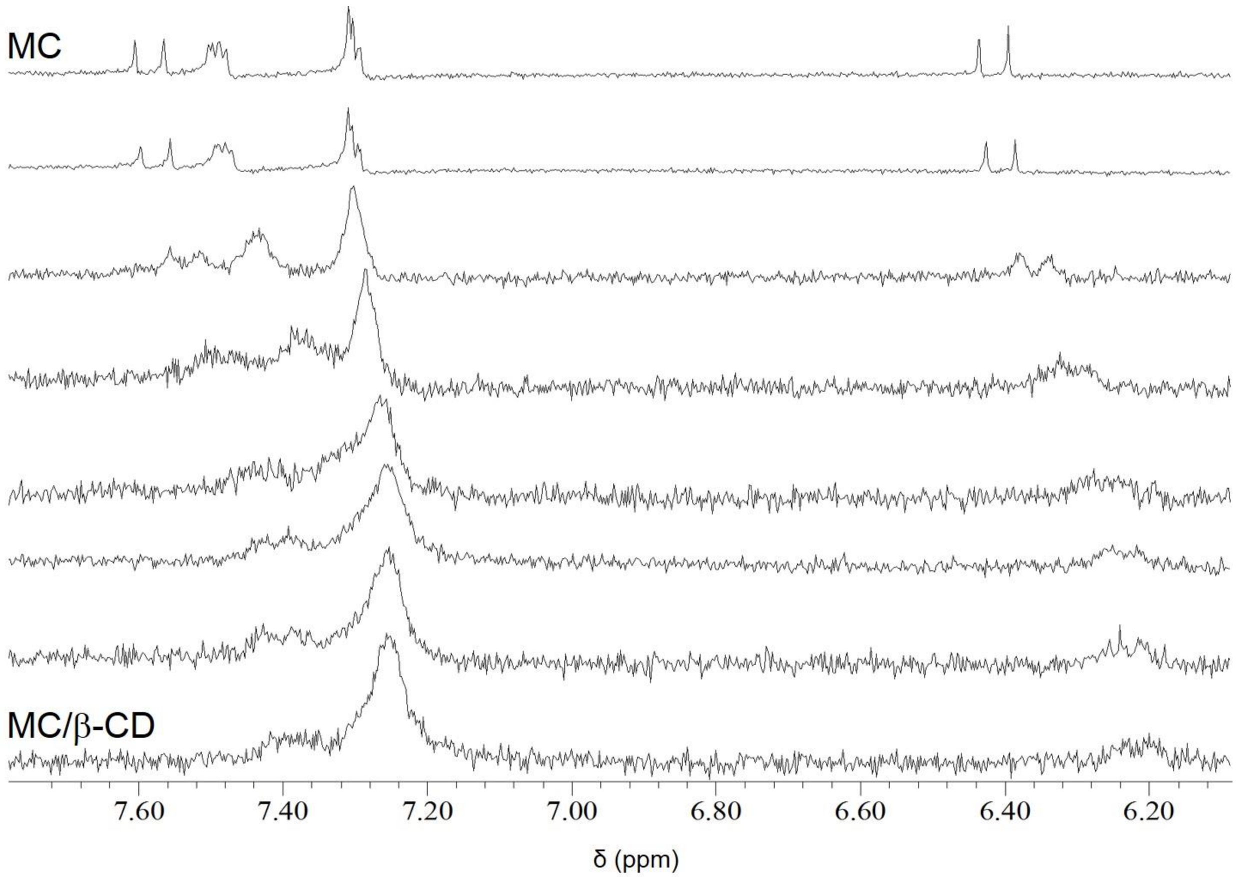

Figure S10. ^{1}H NMR (400 MHz) titration of *E*-MC (0.6 mM) with β-CD (0−15 equivalents) in D_2O at pD 7.

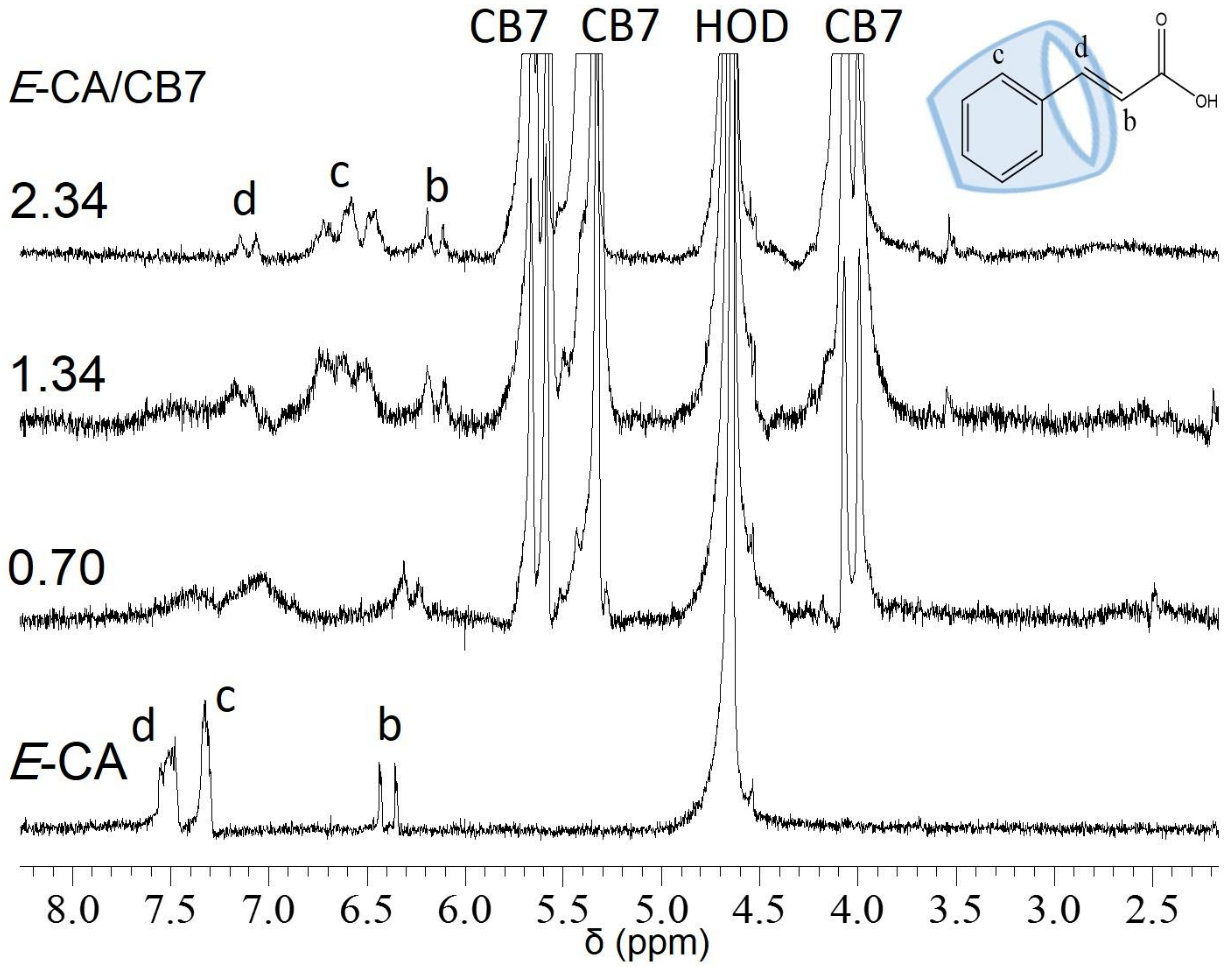

Figure S11. ^{1}H NMR (400 MHz) titration of *E*-CA (3.25 mM) with CB7 (0−2.34 equivalents) in D_2O at pD 2.5.

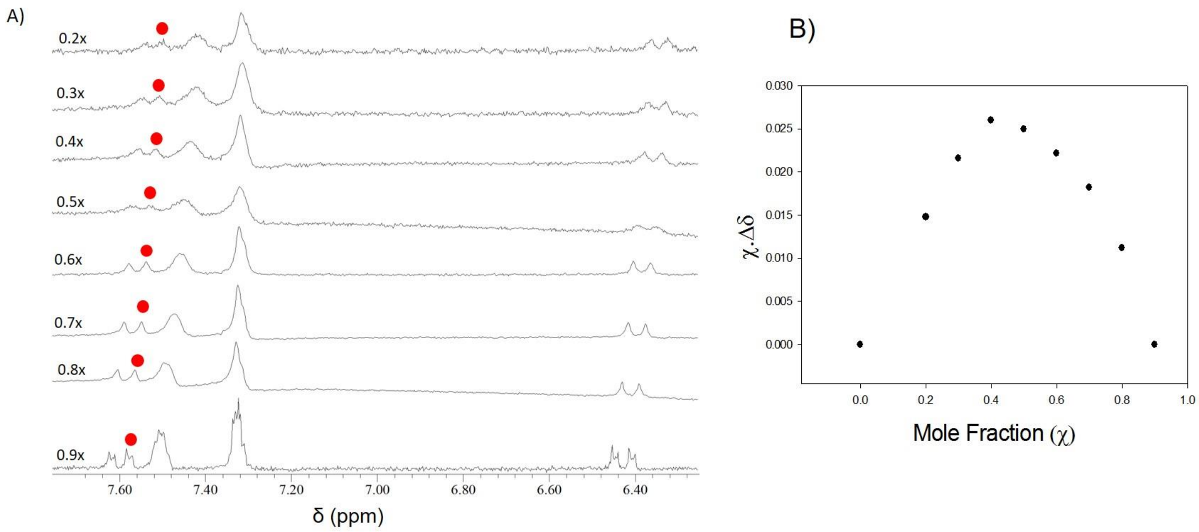

Figure S12. (A) ^{1}H NMR spectra (400MHz, 2 mM = [MC] + [β-CD]), from which the peak at 7.54 ppm in the bottom spectra was monitored; and (B) Job Plot constructed from the data in part (A).

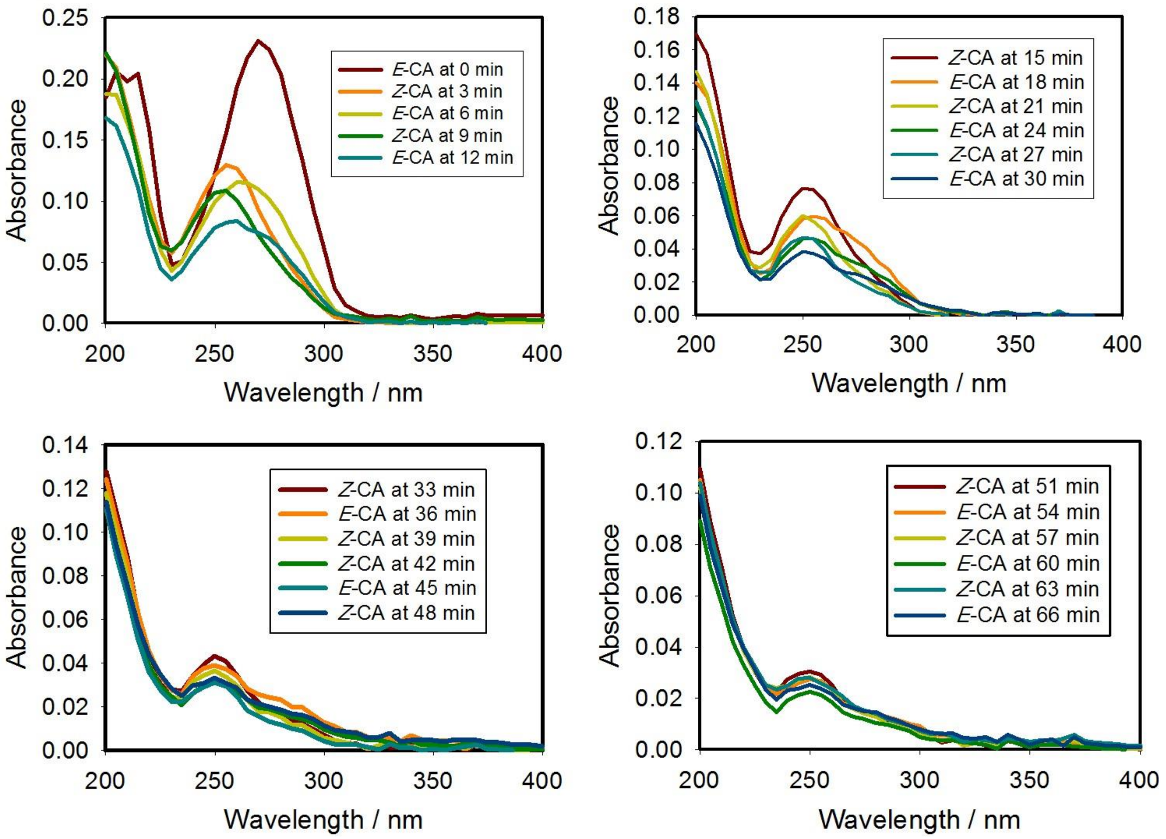

Figure S13. Absorption spectra upon repeated exposure of UV light (300 and 254 nm) to an aqueous solution of CA (16 µM) at pH 5.5 and 298 K as a function of exposure time (each isomer was exposed to irradiation for 3 min at each run) in the absence of CB7.

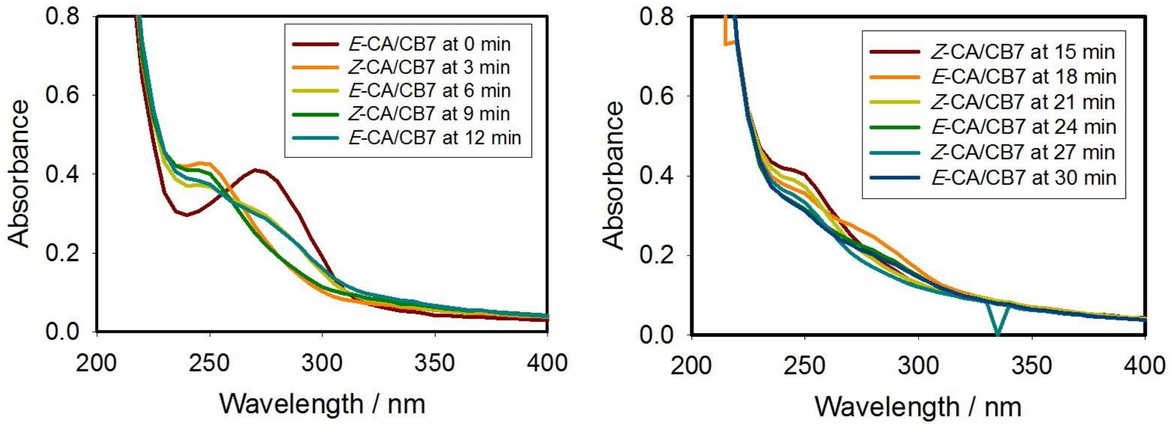

Figure S14. Absorption spectra upon repeated exposure of UV light (300 and 254 nm) to an aqueous solution of CA (32 μM) at pH 5.8 and 298 K as a function of exposure time (each isomer was exposed to irradiation for 3 min at each run) in the presence of CB7 at 1 mM concentration.

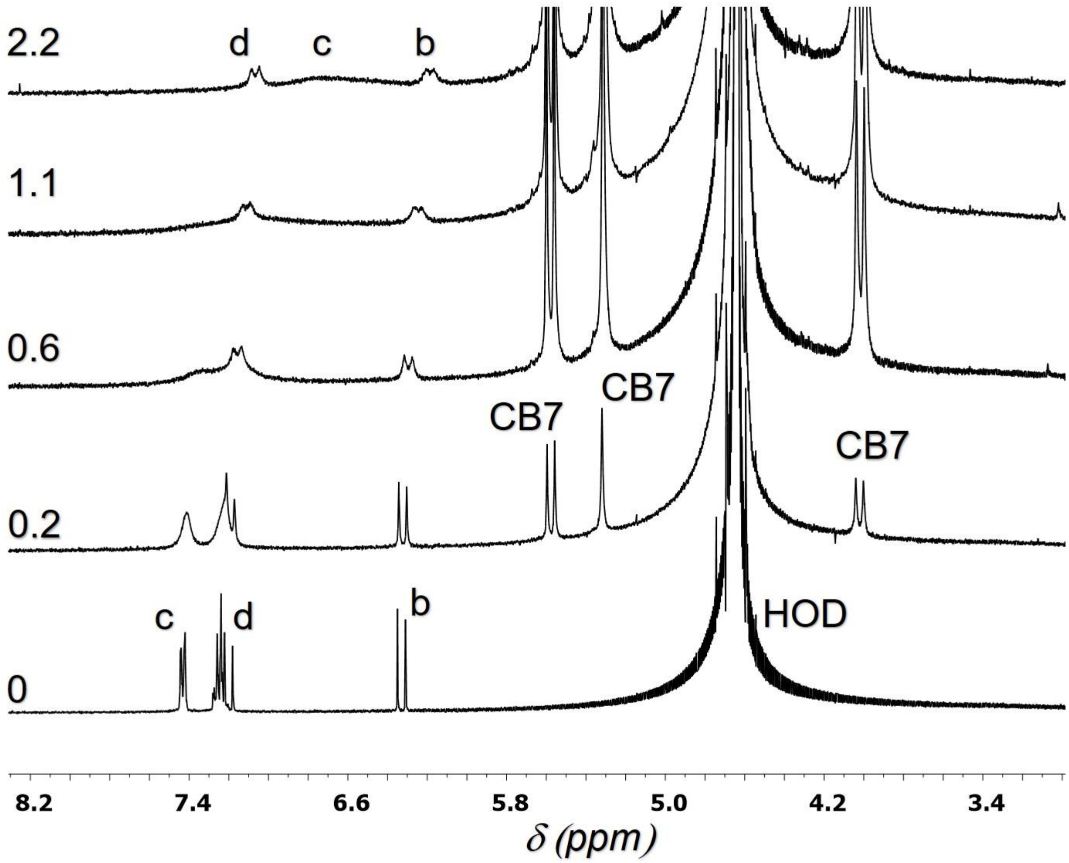

Figure S15. ^{1}H NMR spectra of *E*-CA (1.62 mM) with CB7 (0–2 equivalents) in D_2O (pD 6) at 298 K (400 MHz). Solvent and CB7 peaks are indicated.

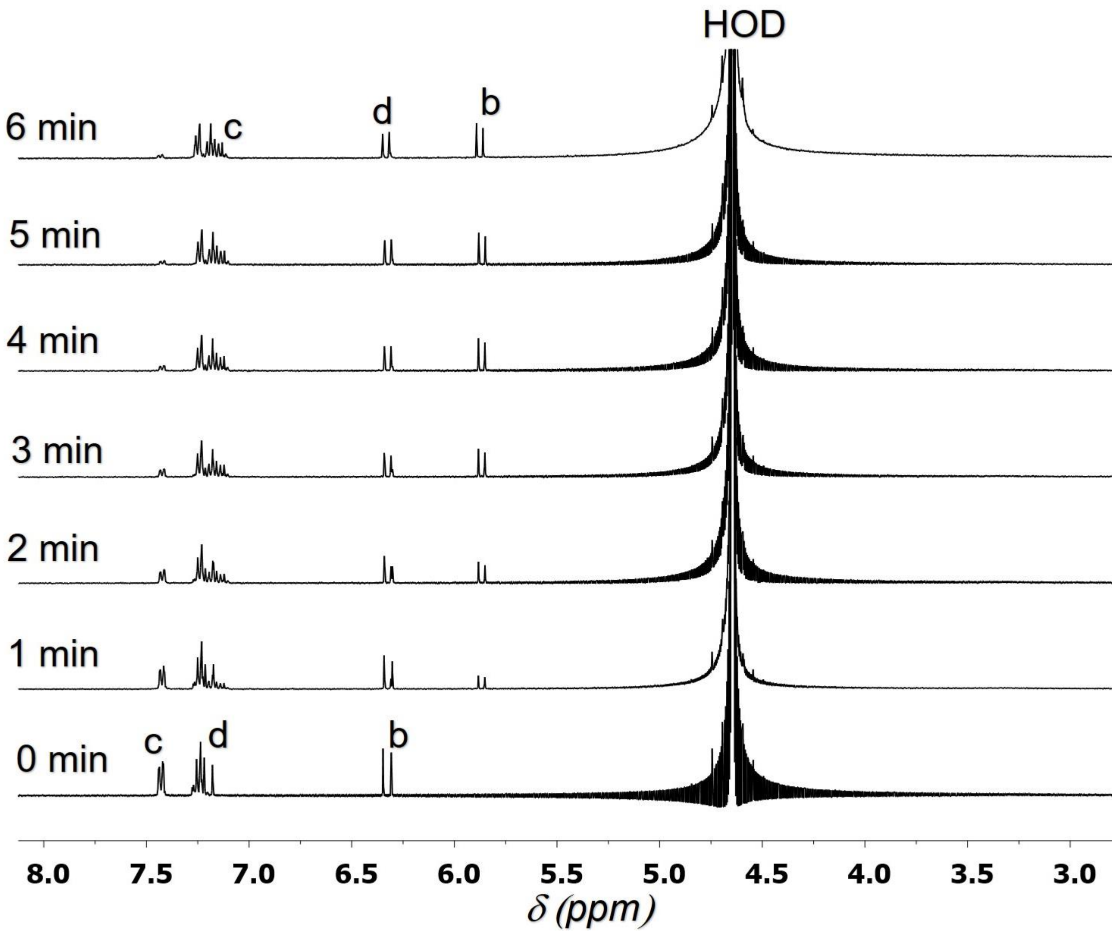

Figure S16. ^{1}H NMR spectra of *E*-CA (1.62 mM) in D_2O (pD 6) at 298 K before and after irradiation with UV light (300 nm) up to 10 minutes (400 MHz). Solvent peak is indicated.

Table S1. The measured percentages associated with the *E* to *Z* photoisomerization upon irradiation of 300 nm to a solution of *E*-CA (1.62 mM) in D_2O at pD 6.

Irradiation time (min)	*E* form (%)[a]	*Z* form (%)[a]
0	100	0
1	39	61
2	37	63
3	23	77
4	20	80
5	11	89
10	11	89

[a]The percentage was calculated by integration of ^{1}H NMR signals.

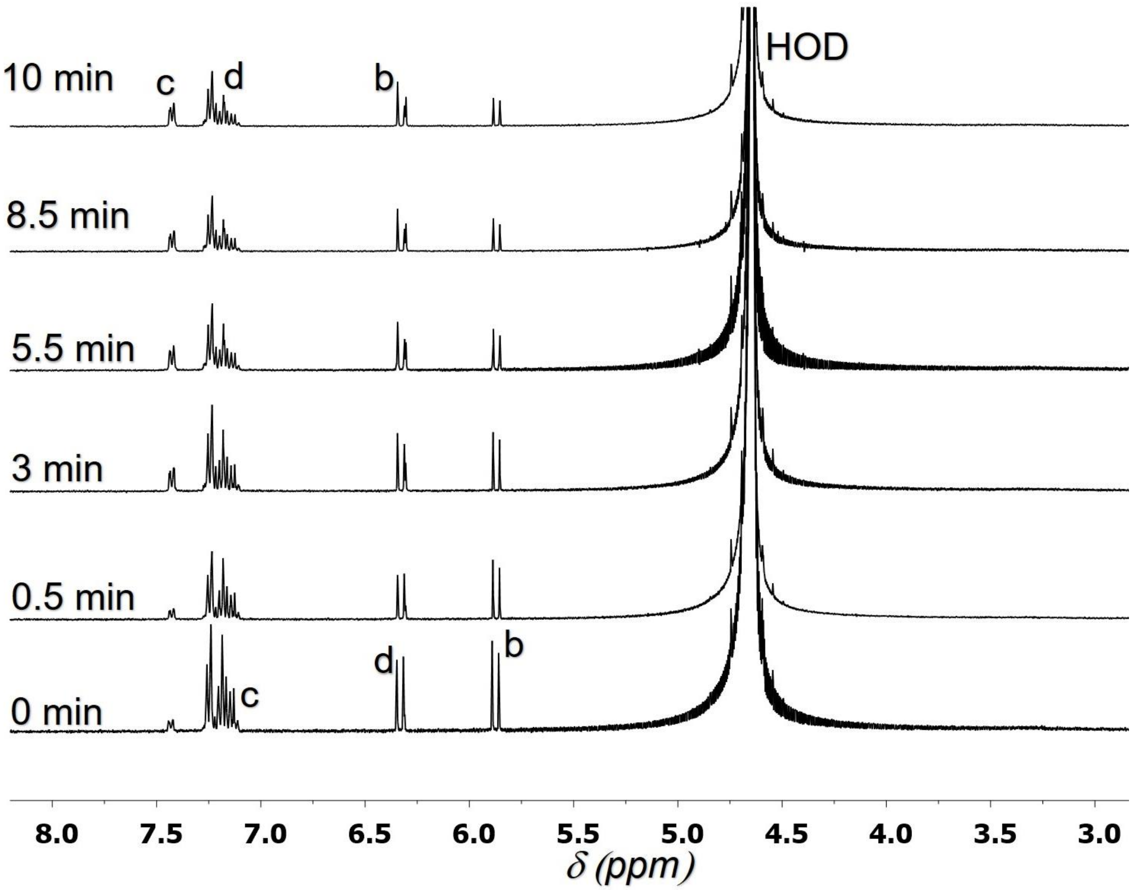

Figure S17. ^{1}H NMR spectra of a 9:1 mixture of *Z*-CA and *E*-CA (total concentration of 1.62 mM) in D_2O (pD 6) at 298 K before and after irradiation with UV light (254 nm) up to 10 minutes (400 MHz). Solvent peak is indicated.

Table S2. The measured percentages associated with the *Z* to *E* photoisomerization upon irradiation of 254 nm to a mixture of *Z*-CA isomer and *E*-CA (*Z*-CA:*E*-CA = 9:1) isomer in D_2O at pD 6.

Irradiation time (min)	*E* form (%)[a]	*Z* form (%)
0	11	89
0.5	23	77
3	29	71
5.5	31	69
8.5	42	58
10	42	58

[a]The percentage was calculated by integration of ^{1}H NMR signals.

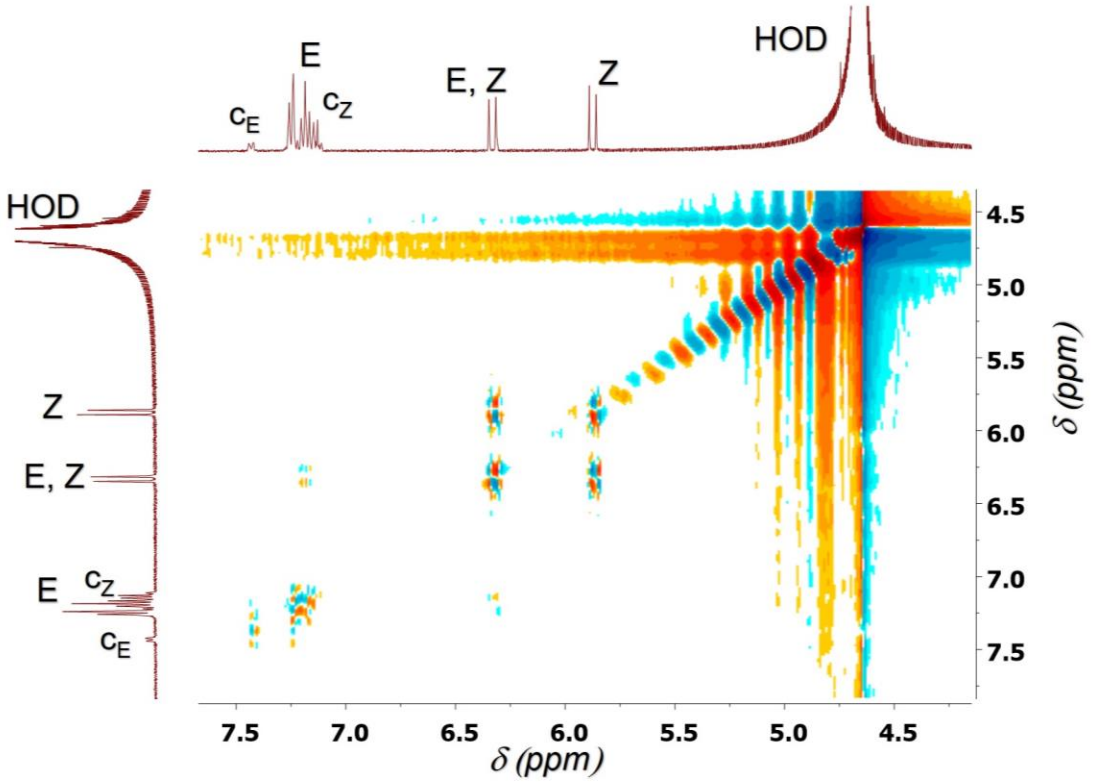

Figure S18. ^{1}H NMR COSY spectrum of a 9:1 mixture of *Z*-CA and *E*-CA (total concentration of 1.62 mM) in D_2O (pD 6) at 298 K (400 MHz).

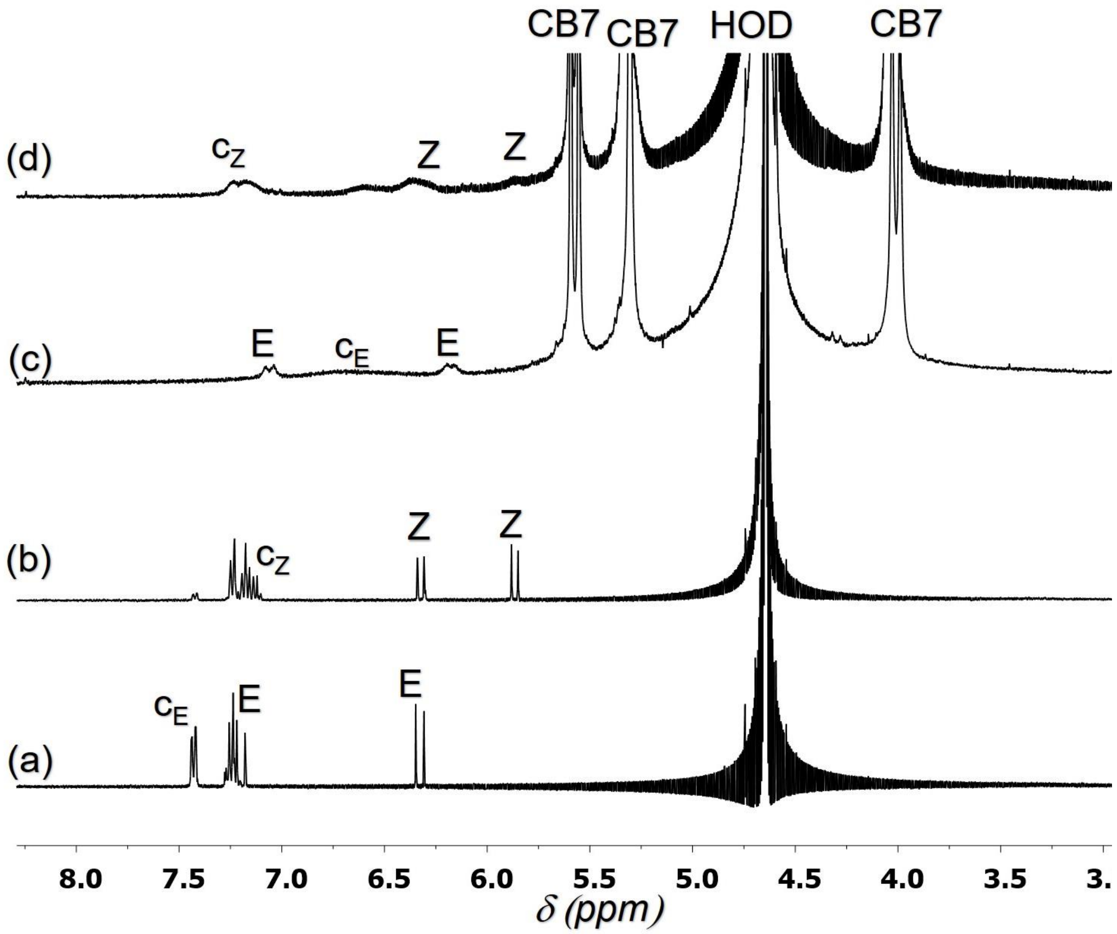

Figure S19. ^{1}H NMR spectra of (a) *E*-CA (1.62 mM), (b) after irradiation of UV light (300 nm) to (a) for 5 min, (c) *E*-CA/CB7 (1.62 mM for *E*-CA and 3.46 mM for CB7), and (d) after irradiation of UV light (300 nm) to (b) for 5 min in D_2O (pD 6) at 298 K (400 MHz). Solvent and CB7 peaks are indicated.

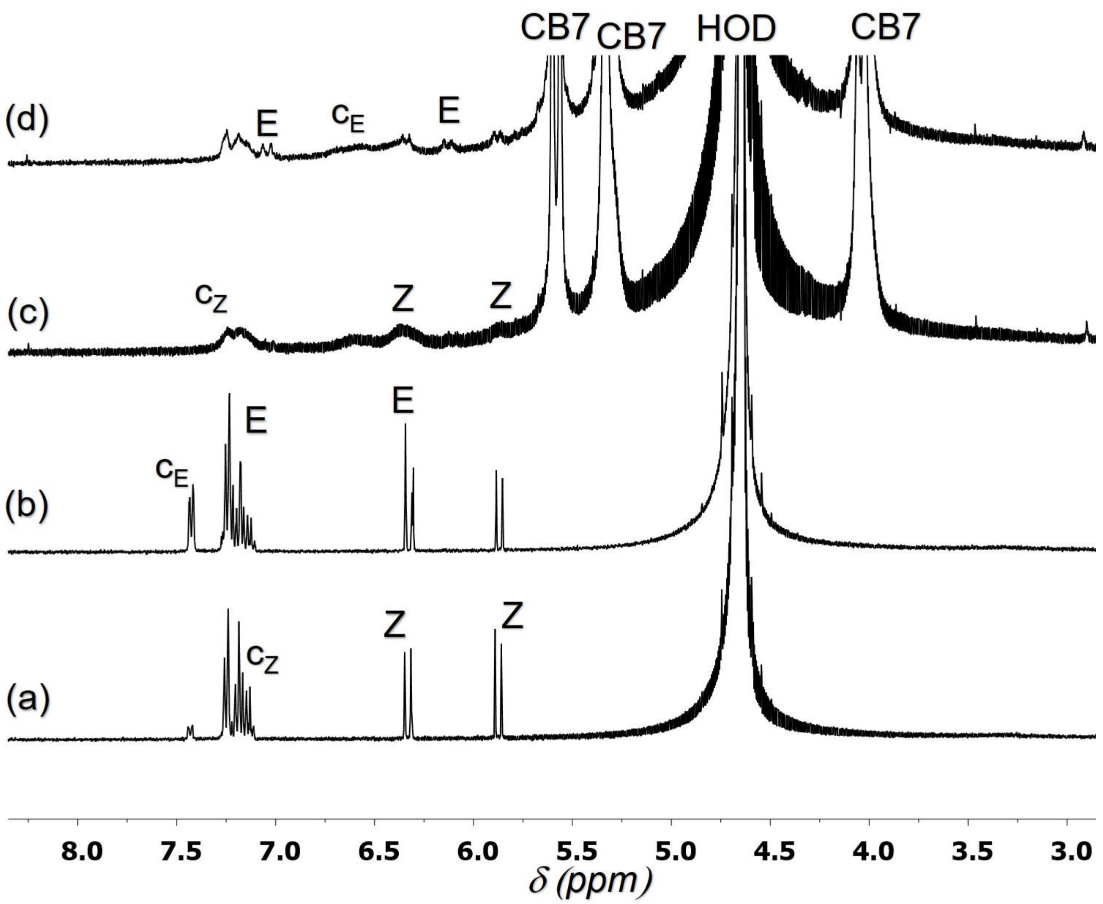

Figure S20. ^{1}H NMR spectra of (a) a 9:1 mixture of *Z*-CA and *E*-CA (total concentration of 1.62 mM), (b) after irradiation of UV light (254 nm) to (a) for 10 min, (c) a mixture of Z-CA and *E*-CA/CB7 (total concentration of 1.62 mM for CA and 3.46 mM for CB7), and (d) after irradiation of UV light (254 nm) to (b) for 10 min in D_2O (pD 6) at 298 K (400 MHz). Solvent and CB7 peaks are indicated.

Table S3. Absorbances (Abs.) data of *E*-CA (16 μM in water and 32 μM in CB7) at 276 nm in the absence and presence of CB7 (1 mM) associated with the *E* to *Z* photoisomerization upon irradiation of UV light (300 nm).

Irradiation time (min)	*E* form (%)[a]	Abs. of *E*-CA	Abs. of *E*-CA/CB7
0	100	0.224	0.405
1	39	0.110	0.270
2	37	0.100	0.260
3	23	0.076	0.229

The measured percentages from Table S1.

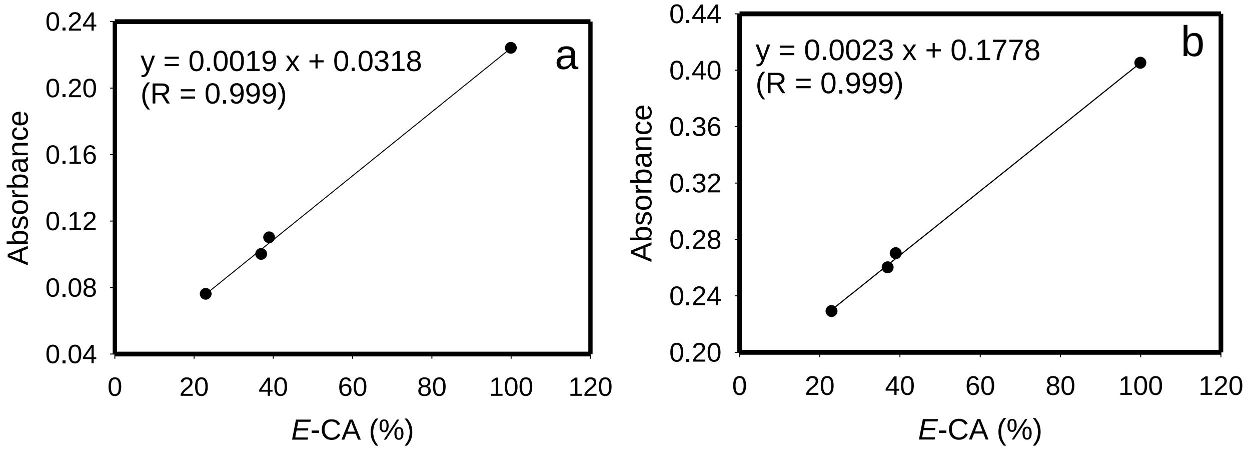

Figure S21 Calibration curves for (a) *E*-CA, and (b) *E*-CA/CB7 complex plotted using the values in Table S3.

Table S4. The calculated percentages of *E*-CA and *E*-CA/CB7 associated with alternating irradiation of 300 nm (3 min) and 254 nm (3 min) from the absorbances (Abs.) data in Figures S13 and S14.

Irradiation wavelength (nm)	*E* (%)	*E*-CA/CB7 (%)	Abs. of *E*-CA	Abs. of *E*-CA/CB7
-	100	100	0.224	0.405
300	23	22	0.076	0.229
254	36	50	0.101	0.294
300	15	19	0.060	0.221
254	20	47	0.070	0.286
300	2	20	0.036	0.224
254	7	36	0.045	0.260
